# Occurrence and environmental risk assessment of pharmaceuticals in the Mondego river (Portugal)

**DOI:** 10.1016/j.heliyon.2024.e34825

**Published:** 2024-07-18

**Authors:** Danijela Kötke, Juergen Gandrass, Célia P.M. Bento, Carla S.S. Ferreira, António J.D. Ferreira

**Affiliations:** a Helmholtz-Zentrum Hereon, Institute of Coastal Environmental Chemistry, Organic Environmental Chemistry, Geesthacht, 21502, Germany; b Research Centre for Natural Resources, Environment and Society (CERNAS), Agrarian Technical School, Polytechnic Institute of Coimbra, P-3040-316, Coimbra, Portugal; c Department of Physical Geography and Bolin Centre for Climate Research, Stockholm University, SE-106 91, Stockholm, Sweden; d Wageningen Environmental Research, Wageningen UR, 6708 PB, Wageningen, the Netherlands

**Keywords:** Pharmaceuticals, UHPLC-MS/MS, Environmental risk assessment, Freshwater, Saltwater, Mondego river, Portugal

## Abstract

In this case study pharmaceuticals were analysed in the Mondego river (Portugal) and their environmental risk assessed by means of risk quotients based on an extensive retrieval of ecotoxicological data for freshwater and saltwater species. The Mondego river crosses Coimbra, the most populated city in the Portuguese Centro Region hosting a complex of regional hospitals. Environmentally relevant and prioritised pharmaceuticals were investigated in this study and their potential hazards were evaluated by conducting a separate risk assessment for the freshwater and estuary parts of the examined river section.

A target analysis approach with method detection limits down to 0.01 ng L^−1^ was used to determine pharmaceuticals. Twenty-one prioritised target analytes out of seven therapeutical classes (antibiotics, iodinated X-ray contrast media (ICM), analgesics, lipid reducers, antiepileptics, anticonvulsants, beta-blockers) were investigated by applying ultra-high pressure liquid chromatography coupled to a triple quadrupole mass spectrometer equipped with an electrospray ionisation source.

The relative pattern of pharmaceuticals along the middle to the lower Mondego showed a quite uniform picture while an approximately 40fold increase of absolute concentrations was observed downstream of the wastewater treatment plant (WWTP) discharge of Coimbra. The most frequently measured substance groups were the ICM, represented by the non-ionic ICM iopromide (β_min_: 3.03 ng L^−1^ - β_max_: 2,810 ng L^−1^). Environmentally more critical substances such as carbamazepine, diclofenac, and bezafibrate, with concentrations up to and 52.6 ng L^−1^, 59.8 ng L^−1^, and 10.2 ng L^−1^ respectively, may potentially affect aquatic wildlife. Carbamazepine revealed elevated risk quotients (RQs >1) along the middle and lower Mondego with a maximum RQ of 53 downstream of Coimbra. Especially for saltwater species, carbamazepine and clarithromycin pose high potential risks.

Especially in periods of low water discharge of the Mondego river, other pharmaceuticals as diclofenac and bezafibrate may pose additional risks downstream of the WWTP.

## Highlights

• WWTP of Coimbra is a major source of pharmaceuticals in the Mondego river.
• Up to 40-fold increase of X-ray contrast media concentrations downstream of WWTP.
• Elevated risk potentials for carbamazepine, bezafibrate, diclofenac, erythromycin.
• Carbamazepine and clarithromycin pose potential adverse effects in the estuary.

## Graphical abstract

**Figure:**
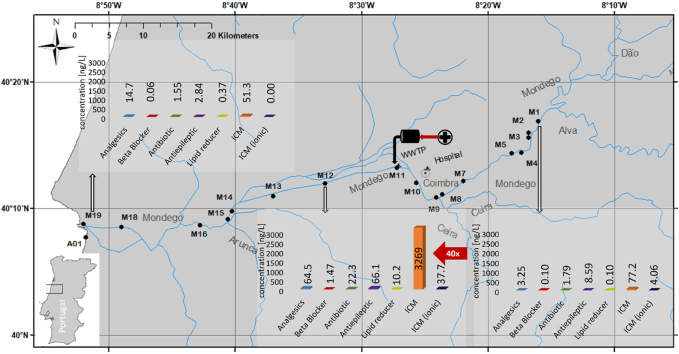

## 1 Introduction

The European Commission authorised over 12,000 products of active substances for human and veterinary use [1]. Since 2006, for the marketing authorisation of human pharmaceuticals an environmental risk assessment is required demonstrating that they do not represent any risk to the environment. These applications are evaluated by the European Medicines Agency (EMA) focusing on the effect of these substances on three trophic levels of aquatic organisms (fish, Daphnia and algae). For substances launched before 2006, those requirements do not apply [2]. Due to the demographic changes in the population, the use, production, and development of pharmaceutically active substances progressed. In 2021 the global pharmaceutical market reached an annual revenue of 1,420 billion U.S. dollars, continuing the increasing global trend [3].

Pharmaceuticals are used in households and hospitals from where they enter the sewage cycle if not discharged untreated into receiving water bodies. Wastewater treatment plants (WWTPs) are the receiving systems of pharmaceuticals and thus are main emission sources into the aquatic environment [4]. Rogowaska and Zimmermann [5] reviewed scientific articles between 2012 and 2021 deducing that the main disposal practise of unnecessary drugs is through household waste or flushing into the sewage water. Consequently, they conclude that a reduction of drug consumption, especially of the over-the-counter pharmaceuticals, and a functioning waste management system is essential.

The proportion of hospital wastewater that contributes to the pharmaceutical load in WWTPs varies between 2 and 20 % [[6], [7], [8]]. This questioned the demand for hospital wastewater treatment prior to introducing the sewage to the WWTPs [9]. Taking the current development of shorter hospitalisation and outpatient care into account a cost intensive hospital wastewater pre-treatment is claimed to be ecologically and economically not justified. Factors affecting the elimination efficiency of WWTPs are related to their technical configurations, as well as the seasonal fluctuating composition of the wastewater influent [10,11]. WWTPs are able to reduce, but not totally eliminate all introduced contaminants and their transformation products [12,13]. Since drugs are often consumed on a regular basis, the effluent represents a mixture which is continuously emitted and, therefore, develops a pseudo-persistent character [14,15]. Transformation products are formed at different elimination stages in the wastewater treatment process. The knowledge of potential transformation products and their ecotoxicological impact is scarce [[16], [17], [18],^19^,^20^].

Studies on the occurrence of pharmaceuticals in aquatic environments covered a limited number of different pharmaceuticals and transformation products [21,22]. Knowledge on ecotoxicologically relevant substances and their transport to estuaries, the coastal and marine environment is still limited [[23], [24], [25], [26], [27]]. Risk assessment studies of pharmaceuticals are scarce especially for brackish and marine waters and different evaluation approaches are followed [2,[28], [29], [30]]. We followed the EU Technical Guidance Document on Risk Assessment [31] which advises for the risk assessment for substances in the estuarine and marine environment to use toxicological data for “ecologically relevant saltwater species”.

In a Portuguese nationwide monitoring exercise for pharmaceuticals in WWTPs, Pereira et al. [32] prioritised six rivers in Portugal, among them the Mondego river, for monitoring programmes. The Mondego is characterised by a large seasonal variation of water discharge [33]. The city of Coimbra with a complex of regional hospitals is located downstream of the sparsely populated upper catchment. Its WWTP effluents enter the Mondego at Choupal further downstream.

This study investigates the presence and potential environmental impacts of prioritised environmentally relevant pharmaceuticals of different therapeutic classes [34,35] in the Mondego river.

## 2 Materials and methods

### 2.1 Study area

**Fig. 1:**
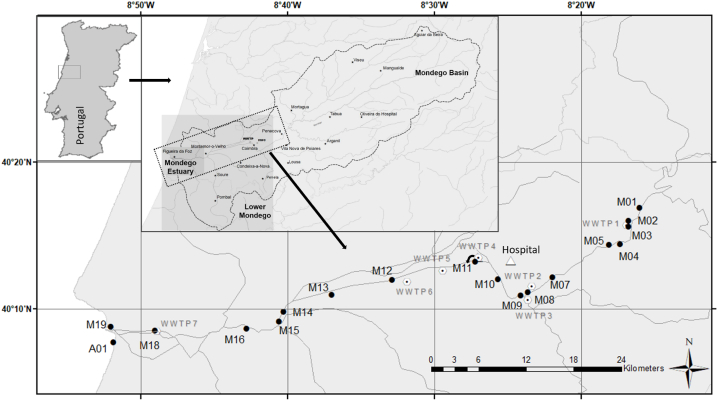
Sampling sites in the lower Mondego catchment including the estuary (M18, M19) and Atlantic Ocean (A01). September 2018 (Software: Esri ArcGIS Desktop 10.8.1); ● sampling points; ⨀ wastewater treatment plant.

The Mondego river was investigated in this study (Fig. 1). The Mondego river catchment is located in the central region of Portugal with an area of 6,670 km^2^, which is divided into three hydro-morphologic subareas: the mountainous Upper Mondego, the Middle Mondego and the agriculturally influenced Lower Mondego [36]. The largest cities along the river are Coimbra (approx. 140,000 inhabitants) and Figueira da Foz (59,000 inhabitants), the latter located at the Mondego estuary. Coimbra represents about 36 % of the population in the catchment area [37] and hosts a complex of regional hospitals. The Mondego river is receiving water inflows from the tributaries Dão, Ançã, Foja, Alva, Ceira, Cernache, Ega, Arunca and Pranto [38]. Water discharge varies largely from less than five m³s^−1^ during drought to more than 600 m³s^−1^ in periods of rain events as measured at Açude Ponte Coimbra [33].

### 2.2 Sampling

Water sampling was performed within three days in September 2018 with an average water discharge at Açude Ponte Coimbra of 16 m³ s^−1^ [33]. The sampling started close to Vila Nova, a village in the municipality of Penacova, ending downstream in the city Figueira da Foz where the river Mondego reaches the Atlantic coast (Fig. 1, Supplementary Table S1). At 18 stations close to the riverbanks or coastline, 2 L-water samples were taken in pre-cleaned polypropylen (PP) screw cap bottles after rinsing twice with sample water. For quality control, field blanks and at a few stations triplicate water samples were collected. At all stations parameters such as salinity, pH-value and temperature were measured *in loco* with a Multi 3420 (WTW, Germany) (Supplementary Table S1). After sampling, samples were stored at the Higher Education School of Agriculture in Coimbra in the dark at −20 °C. The frozen water samples were transported to the Helmholtz-Zentrum Hereon in Geesthacht with a specialist carrier ensuring that the samples stayed frozen until analysis.

### 2.3 Selected pharmaceuticals

This study investigates 21 chemical compounds from 7 pharmaceutical classes (Table 1). These pharmaceuticals were selected according to a prioritisation of Bergman et al. [34] and applied by Kötke et al. [35] in an environmental risk assessment in the German Bight and Baltic Sea area.

**Table 1:** Analysed pharmaceuticals and pharmaceutical classes.

Class	Compound	CAS no.	Pharmaceutical class
Antibiotics	Erythromycin	114-07-8	Macrolide
	Clarithromycin	81103-11-9	Macrolide
	Roxithromycin	80214-83-1	Macrolide
	Sulfamethoxazole	723-46-6	Sulfonamide
	Sulfamethazine	57-68-1	Sulfonamide
	Sulfadimethoxine	122-11-2	Sulfonamide
	Tiamulin	55297-95-5	Pleuromutilin
	Lincomycin	154-21-2	Lincosamide
ICM a	Iomeprol	78649-41-9	Non-ionic ICM
	Iopamidol	60166-93-0	Non-ionic ICM
	Iopromide	73334-07-3	Non-ionic ICM
	Amidotrizoic acid	117-96-4	Ionic ICM
	Ioxitalamic acid	28179-44-4	Ionic ICM
	Iohexol	66108-95-0	Non-ionic ICM
Analgesics	Paracetamol	103-90-2	Analgesics and antipyretics
	Diclofenac	15307-86-5	Nonsteroidal anti-inflammatory drug
Lipid reducer	Bezafibrate	41859-67-0	Lipid-lowering agent
Antiepileptic	Carbamazepine	298-46-4	Anticonvulsant
Anticonvulsant	Primidone	125-33-7	Anticonvulsant
Beta-Blocker	Nadolol	42200-33-9	Beta blocker
	Propranolol	525-66-6	Beta blocker

a Iodinated x-ray contrast media.

#### 2.3.1 Antibiotics

In 2014, in Portugal 254 t of antibiotics have been consumed, 30 % for human and 70 % for veterinary use [39]. In recent years, overall sales of veterinary antimicrobials dropped, tetracylines and sulphonamides being the most widely used antibiotic classes [40]. This declining trend is presumably due to restrictions in the EU for the non-therapeutic use of antibiotics in animal husbandries [41] and the rising awareness of the risk of multiresistent pathogens [40,42].

#### 2.3.2 ICMs

The pharmaceutical group of iodinated X-ray contrast media (ICM), with its main representatives iomeprol, iopamidol and amidotrizoic acid, constitute a class of poorly degradable substances. Their physicochemical properties such as polarity, stability and lack of biodegradability brands them suitable for the application in the diagnostic imaging. Thus, and due to their polar character, 90–100 % of the ICM are excreted unmetabolised by the human body within 24 h [43,44]. But the properties of these substances are linked with disadvantages reported in the elimination rates close to 0 % in conventional WWTPs [45,46]. Enhanced wastewater treatment systems using ozonation could improve the removal efficiency e.g. for iopromide (<50 %) [47], but within this transformation process by-products with potentially higher bioactivity could be formed [[48], [49], [50], [51]]. When treating water for drinking purposes with chlorine or chloramine disinfection in the presence of ICM such as iopamidol, iopromide and iohexol, an enhanced cytotoxicity and genotoxicity could be observed, possibly affecting public health [52,53].

#### 2.3.3 Analgesics

According to Pena et al. [54] anti-inflammatories are the most-frequently sold drugs, but not the most consumed ones in the OECD countries of Asia, North American and Europe. Further, they were the most studied pharmaceutical group for the removal in WWTPs. Miege et al. [46], Gaffney et al. [55] and Sadutto et al. [56] showed for analgesics such as paracetamol removal efficiencies close to 100 % in WWTPs with secondary treatment. In this context, the occurrence in receiving water bodies leads to the assumption that there must be other input pathways from untreated wastewater [57].

#### 2.3.4 Others

Bezafibrate, a drug from the group of lipid regulator, has comparably high consumption rates varying from 1,574 (Switzerland) to 39,158 kg per year (Germany) in 2010, depending on the number of residence [21]. Approximately 95 % of the administered dose is excreted via urine within 48 h, 50 % as unchanged bezafibrate and the rest as decomposition products [58]. The average removal efficiency in WWTPs is 40 %, this average rate includes data from 52 publications reviewed by Pereira et al. [21].

The β-adrenergic receptor blocking compounds (β-blockers) propranolol and nadolol are drugs used mainly for the treatment of cardiovascular diseases. Globally, an increase of 7.13 % per year is expected on the β-blockers market [59]. Yi et al. [60] reviewed the class of β-blockers regarding distribution, transformation and ecotoxicity. In various environmental matrices β-blockers could be detected and are showing a wide range of removal efficiency in WWTPs from 0 % to 99 %. Especially propranolol was highlighted as the substance among the β-blockers with the highest toxicity.

In the class of antiepileptic drugs primidone and carbamazepine show low biological degradation and sorption resulting in low removal rates in WWTPs with less than 25 % [21,61]. Since these drugs are targeting the central nervous system their biological effects in nontarget organisms may pose risks for higher trophic levels and effect the biological ecosystem. Mezzelani et al. [62] compiled an overview of those biological effects on marine organisms, presenting the diverse impact of e.g. carbamazepine on bivalves showing oxidative stress, activation of immune responses, affected embryo development with shell malformations and neurotoxicity.

### 2.4 Chemicals and reagents

The analytical setup was developed based on native and mass labelled standards with the highest purity available (>98 %). Methanol hypergrade for LC-MS LiChrosolv® (Merck, Darmstadt, Germany) was used for the liquid chromatography and as solvent for the solid phase extraction (SPE). Ultrapure water (MilliQ-water) was provided by a Milli-Q Integral 5 system (TOC <3 ppb and R = 18.2 MΏ) from Merck. Formic acid, as additive for the mobile phase, was supplied by Sigma-Aldrich (Traufkirchen, Germany), and hydrochloric acid (HCl) suprapur®, used for pH adjustment, was supplied by Merck. All chemicals and reagents are described in detail in Kötke et al. [35].

### 2.5 Sample preparation and analysis

Sample preparation and analysis followed the procedure described in Kötke et al. [35]. Briefly, the water samples were thawed and filtered through glass microfiber filters (GF/F, Ø 47 mm, Whatman, GE Healthcare). One 1 L aliquot was adjusted to pH 2 using hydrochloric acid for the quantification of acidic compounds (amidotricoic acid, ioxitalamic acid). Another 1 L aliquot remained at the original pH-value (range of 6.9–8.6, Supplementary Table S1). Prior to enrichment, the aliquots were spiked with mass-labelled internal standards. The enrichment (Factor 1333) was performed with Oasis HLB cartridges (Hydrophilic-lipophilicbalanced reversed-phase sorbent, 60 mm, 500 mg, 6 cc, Waters, Milford, MA, USA). After a washing step with MilliQ-water, the cartridges were eluted with methanol and concentrated to 150 μL applying a gentle heated nitrogen flow. The final extracts for the quantification of the pharmaceuticals were set up to a final volume of 750 μL by adding the injection standard (Benzotriazole-D4) and MilliQ-water and filtrated through a nylon syringe. The analytical system consisted of an Agilent Technologies 1290 UHPLC coupled to an Agilent Technologies 6490 triple quadrupole mass spectrometer operating with the Agilent Jet Stream electrospray ionisation (ESI) source. Data analysis was carried out using the software MassHunter B08.00 QQQ Quantitative Analysis. The method quantification limits for surface water varied from 0.01 to 0.65 ng L^−1^ for most of the analytes except for the ICM iohexol (2.28 ng L^−1^) and ioxitalamic acid (3.77 ng L^−1^) and the antibiotic sulfamethazine (1.28 ng L^−1^). Further details on LC-MS/MS parameters, MRM transitions, recoveries and method quantification limits can be found in the supplementary (Supplementary Tables S2–S5).

In the present study, generally, 12-point calibration curves with calibration standards ranging from 0.05 ng mL^−1^ to 500 ng mL^−1^ were generated. The native standards in the required concentrations, the labelled internal standards and the injection standard were set up to a final volume of 750 μL in 20/80 Methanol/MilliQ. The calibration curves with a weighing factor of 1/x were linear with regression coefficients of R^2^ > 0.99. For carbamazepine a lower calibration range (up to 100 ng mL^−1^) was selected since signal saturation could be observed. Further, it was necessary to extend the calibration range for the ICM iopromide by the addition of three higher concentration levels (1,000, 3,000 and 5,000 ng mL^−1^), in order to quantify the iopromide concentration at sampling point M12.

For quality control, standard controls and blank injections, field blanks as well as triplicate water samples from stations M11 and A01 were analysed. No carryover between subsequent analyses was observed. Field blanks did not interfere in the quantification and method precision under repeatability conditions was in the range of 0.6 %–12 % (triplicates at station M11), except for paracetamol and clarithromycin with concentrations close to MQLs (maximum 29 % for triplicates at station A01).

### 2.6 Environmental risk assessment

In order to evaluate potential risks of measured concentrations of pharmaceuticals in the Mondego river, datasets of effect concentrations for aquatic organisms for freshwater and brackish/marine species were compiled from databases and publications (Supplementary Table S14). Predicted no-effect concentrations (PNECs) for freshwater species were derived applying assessment factors from Bergmann et al. [34]. For marine/brackish species, PNECs were calculated depending on available effect concentrations for organisms from different trophic levels and resulting assessment factors [63,64]. Assessment factors, starting with 10,000, were lowered to 1,000 when L(E)C50 of three trophic levels (algae, crustaceans and fish – freshwater or saltwater species) AND for two additional marine taxonomic groups (e.g. echinoderms, molluscs) were available.

Risk quotients (RQs) were calculated as quotients of maximum measured environmental concentrations (MECs) and PNECs. For the sections (Supplementary Tables S8–S12) of the Mondego river risk assessment have been conducted by applying the associated MECs and PNECs. These RQs were classified in medium risk for 0.1 ≤ RQ < 1 and in high risk for RQ ≥ 1 [65,66] and used for the environmental risk evaluation.

## 3 Results and discussion

### 3.1 Pharmaceutical concentrations in the Mondego and comparison to other studies

**Fig. 2:**
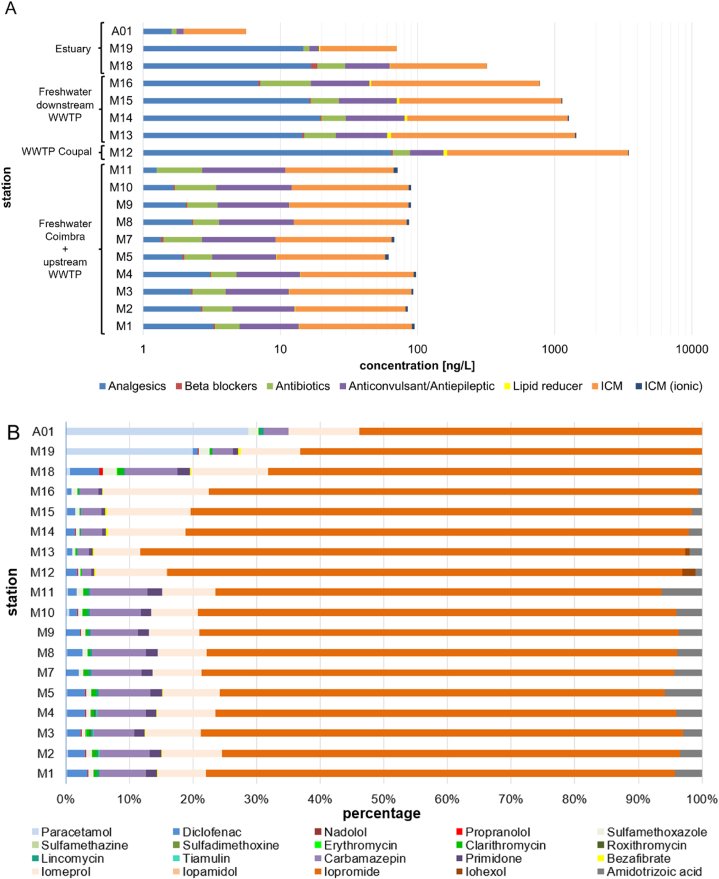
Pharmaceuticals in the Mondego river (September 2018). **A**. Sum concentrations of therapeutical classes. **B**. Shares of individual pharmaceuticals.

Sum concentrations of therapeutical classes and shares of individual pharmaceuticals at individual sampling stations are depicted in Fig. 2 and absolute concentrations are given in Supplementary Table S6. In the middle and lower Mondego (stations M1 - M11), sum concentrations ranged from 60 ng L^−1^ to 100 ng L^−1^. In the lower Mondego (stations M12 - M19), a maximum sum concentration (β _sum_) of 3,500 ng L^−1^ was reached downstream the WWTP discharge of Choupal, in Coimbra city, with a subsequent decrease towards the estuary (β _sum_ = 6 ng L^−1^). The samples upstream of Choupal showed uniform sum concentrations and shares of individual therapeutical classes revealing a pseudo-persistent characteristic obviously due to continuous emissions of the investigated pharmaceutical compounds [67]. The contributions of therapeutical classes to sum concentrations remained approximately constant throughout the Mondego except the estuary with an increase in analgesics due to higher concentrations of paracetamol.

**Fig. 3:**
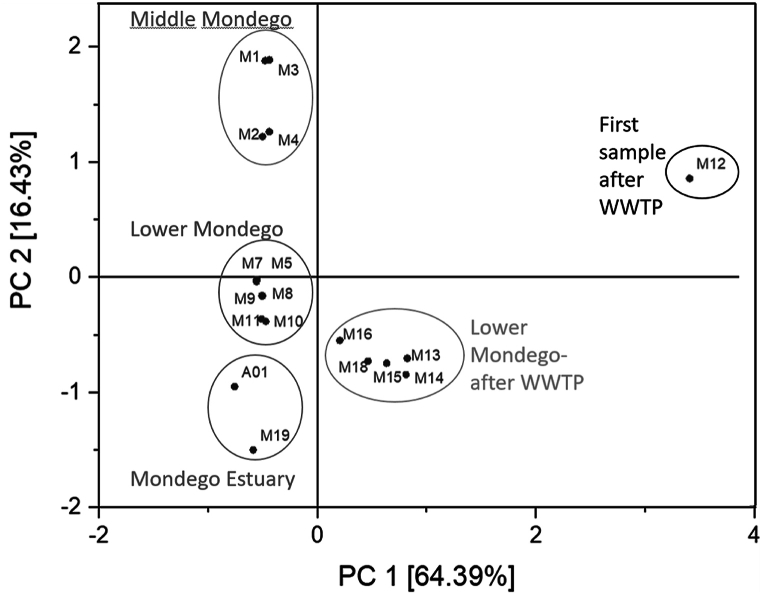
Principal component analysis. Concentrations above limit of detection (MDL) and below limit of quantification (MQL) set to calculated concentrations.

To define coherent areas, a Principal Component Analysis (PCA) was performed (Fig. 3). The PCA underlined the constant pattern for M1 - M4 and M5 - M11, the single event at station M12 and the cluster with all stations following M12 up to the estuary (M13 - M18), and the estuary/coastal region with M19 and A01.

The dominant pharmaceutical class in the Mondego are the ICM, mainly represented by the non-ionic iopromide, accounting for up to 80 % of the total measured pharmaceutical load, and iomeprol (10 %). Their concentrations ranged between 43.1 and 70.9 ng L^−1^ and 5.2–9.0 ng L^−1^ before the WWTPs’ discharge, respectively. The first sampling point (M12) after the discharge showed high pharmaceutical concentrations, for iopromide and iomeprol an increase by a factor of 40–50 to a concentration of 2,810 ng L^−1^ and 386 ng L^−1^, respectively.

The city of Coimbra is in the outlet of the sparsely populated mountain areas. The WWTP of Choupal is receiving municipal wastewaters from the city of Coimbra as well from a complex of central hospitals serving a region with more than 2 million inhabitants [7]. Santos et al. [7] investigated loads of selected pharmaceuticals in wastewaters from hospitals in Coimbra as well as in influents and effluents of the WWTP of Choupal. In their study iopromide was the dominating compound among the investigated pharmaceuticals contributing about 60 % (maximum 80 %) to the sum concentrations of pharmaceuticals in the WWTP effluent in accordance with our findings at M12 downstream of the WWTP discharge. In the WWTP influent 13 % of the iopromide load was stemming from hospitals. The larger contribution of iopromide was originating from households as these hospital-dispensed medicinal products are normally applied in outpatient treatment, and the excretion happens mainly at home [68]. A large reduction of ICM emissions from WWTPs could be demonstrated in a project in Germany in which the patients undergoing radiological investigations were asked to discard their urine in special bags with adsorbing material and to dispose them via domestic waste [69].

The second most frequently detected pharmaceutical compound was the anticonvulsant carbamazepine, with a concentration range of 5.0–7.6 ng L^−1^ upstream of Coimbra, being in the same concentration range as in other European rivers (Supplementary Table S7). Downstream of the WWTP discharge (station M12), the concentration of carbamazepine reached a maximum of 52.6 ng L^−1^, subsequently decreasing towards the estuary. Cunningham et al. [70] reported that the share of unmetabolised carbamazepine in excreted urine is 2 %. Therefore, future studies should incorporate the main metabolites of carbamazepine and their toxicological effects should be evaluated.

The pharmaceutical group of analgesics was ranging between 1.25 ng L^−1^ and 3.25 ng L^−1^ in the first part of the river Mondego (M1 - M11), mainly dominated by the nonsteroidal anti-inflammatory drug diclofenac (90 %) with an increase in concentration by a factor of 30 downstream of Coimbra (M12).

The share of paracetamol was minor, possibly indicating the effective removal of the substance in the wastewater treatment network. This was confirmed in the study by Santos et al. [7], in which a removal efficiency of 99 % was calculated for paracetamol in the WWTP of Choupal. The highest concentration of paracetamol (14.2 ng L^−1^) was unexpectedly found in the estuary at Figueira da Foz (M19) largely influenced by marine water at a measured salinity of 30 psu. Figueira da Foz and the neighbouring coast are a touristic area and highly populated during summer months. The elevated concentrations of paracetamol in the Mondego estuary are in accordance with findings from Lolic et al. [71] who found paracetamol along the coastline of North Portugal with median concentrations of 62.8 ng L^−1^ (*n* = 14). The authors presume that the main source of paracetamol is coming from untreated effluents as well as WWTP discharges.

Sulfamethoxazole and clarithromycin were the major compounds in the group of antibiotics, with a mean share of 54 % (0.09–12.9 ng L^−1^) and 25 % (0.01–4.7 ng L^−1^) with regard to the total antibiotic concentration in the investigated river samples, respectively.

A comparison between our results with concentration ranges of pharmaceuticals of other studies in rivers and coastal waters is given in Supplementary Table S7. Generally, concentrations were in similar but broad ranges due to local characteristics as river discharge, population, point and diffuse sources, country-specific use of medicinal products or seasonal variations.

The Mondego is a highly regulated river with moderate annual water discharges of 100 m³ s^−1^ with lowest discharges during the typical dry summer periods due to less intensive freshwater inflows in summer than in winter [32]. Large seasonal variations in freshwater inflows are the main factor for the varying residence times of 1–12 days for water throughout the Mondego to the Atlantic Ocean [72]. Our sampling campaign took part during three days in September 2018, a period with low water discharge and accordingly high residence times.

Thus, generally lower concentrations of pharmaceuticals than in our study can be expected to occur in the Mondego during the winter season with higher water discharges. To overcome the limitations of our study and include seasonal variations, sampling campaigns at different water discharges throughout the year would have to be conducted.

### 3.2 Environmental risk evaluation

**Fig. 4:**
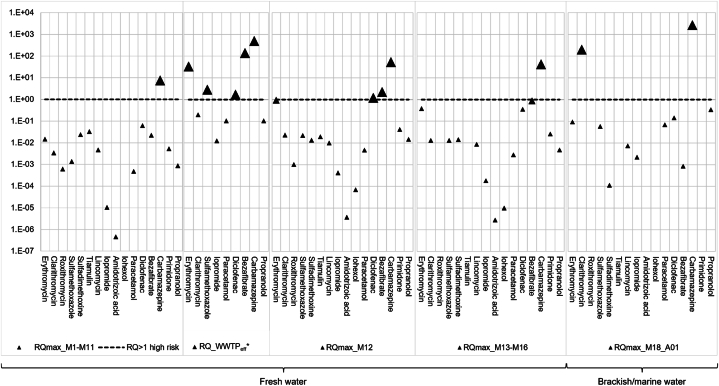
Environmental risk assessment. Risk quotients (RQ) high risk (RQ ≥ 1); medium risk (0.1 ≤ RQ < 1); no risk (RQ < 0.1); *WWTP_eff_ (wastewater treatment plant, effluent) data from Santos et al. (2013).

Environmental Risk Assessment (ERA) was applied following the procedure described in chapter 2.6. ERA was conducted separately for the middle and lower Mondego upstream of Coimbra (Supplementary Table S8), station M12 downstream of Coimbra (Supplementary Table S9), freshwater stations further downstream in the lower Mondego (Supplementary Table S10) and brackish/marine stations (Supplementary Table S11). An overview of all resulting risk quotients (RQs) including RQs derived from concentrations in effluents of the WWTP of Choupal in Coimbra [7] (Supplementary Table S12) is depicted in Fig. 4.

The antiepileptic carbamazepine showed high risk quotients (RQs >1) in all investigated freshwater sections of the Mondego with a maximum RQ of 53 downstream of Coimbra (station M12), and with even higher RQs in the estuary due to comparably very low effect concentrations for marine species, especially for embryo-larval development of sea urchins (RQ up to 2.660). Additionally, at station M12, concentrations of diclofenac, bezafibrate, and erythromycin increased by a factor up to 60, resulting in increased risks for diclofenac (RQ = 1.2) and bezafibrate (RQ = 2.2) and medium risk for erythromycin (RQ = 0.94). Further downstream, concentrations for these compounds were decreasing, presumably due to dilution as well as transformation processes, and thus for diclofenac, bezafibrate, and erythromycin medium risks were achieved before reaching the Mondego estuary. Generally, ecotoxicological data for marine species are scarce implying high assessment factors. Thus, in the

brackish/marine section, for carbamazepine and clarithromycin an assessment factor of 1,000 had to be applied, giving RQs of up to 2,660 (carbamazepine) and 20 (clarithromycin).

The high RQ for carbamazepine in the estuary results from an EC50 for embryo-larval development of the echinoderm *Paracentrotus lividus*. In a weighted approach the RQ would be significantly lower, but still indicating a high risk (Supplementary Table S11).

Following our ERA approach, using the same set of PNECs, we calculated RQs for maximum concentrations of pharmaceuticals in effluents of the WWTP in Choupal found by Santos et al. (2013). Not surprisingly, RQs were higher (up to more than one order of magnitude) compared with RQs downstream the WWTP at M12 in our study (Supplementary Table S8). Although, the WWTP effluents were sampled seven years before our sampling campaign in the Mondego, they showed a similar pattern of high risk pharmaceuticals: carbamazepine, bezafibrate, erythromycin, diclofenac, and additionally sulfamethoxazole (Fig. 4).

A comparison between our study and other recent ERA studies for pharmaceuticals in surface waters is given in Supplementary Table S13. Figuiere et al. [29] conducted an ERA in Swedish surface waters following the EU Guideline [31] as in our approach. Other authors calculated RQs separately for individual trophic levels (algae, crustaceans, fish), and Fonseca et al. [30] calculated potentially affection fractions (PAFs) based on species sensitivity distributions for chronic exposure. Thus, a direct comparison of potentially relevant pharmaceuticals for the Mondego with the situation in other surface waters is only possible to some extent. As in our study, in some of the ERAs carbamazepine, bezafibrate, diclofenac and the antibiotics erythromycin and clarithromycin have been classified as medium or high risk pharmaceuticals for the respective aquatic ecosystems. This is in accordance with Yang et al. [73] who published a critical review dealing with the determination of globally relevant micropollutants. By applying a weighted average risk quotient (WARQ) the aim is followed to achieve a more comprehensive priority determination.

## 4 Conclusions

In the lower and middle Mondego river in Portugal, 19 pharmaceuticals of different therapeutic classes were quantified. The WWTP of Choupal in the city of Coimbra was identified as a major point source resulting in an up to 40fold increase of concentrations of pharmaceuticals in the Mondego about eight km downstream of the WWTP discharge.

The executed environmental risk assessment (ERA) for freshwater species has shown medium to high risk potentials for erythromycin, carbamazepine, diclofenac and bezafibrate. The ERA for marine and brackish species indicated high risk potentials for carbamazepine and clarithromycin, partially due to high assessment factors resulting from scarce toxicological data for marine species. A better set of effect concentrations for marine species would reduce assessment factors and allow an improved ERA.

The results highlight that especially in close vicinity of the discharge of the WWTP in Choupal the aquatic ecosystem of the Mondego can be affected by a number of pharmaceuticals. This applies especially for periods with low water discharge during extended droughts, expected to occur even more frequently in future due to climate change. An improved treatment of the wastewater of the city of Coimbra including its complex of hospitals should be considered.

## Ethical approval

Not applicable.

## Consent to participate

Not applicable.

## Consent to publish

Not applicable.

## Funding

The authors declare that no funds, grants, or other support were received during the preparation of this manuscript.

## CRediT authorship contribution statement

**Danijela Kötke:** Writing – review & editing, Writing – original draft, Methodology, Investigation, Conceptualization. **Juergen Gandrass:** Writing – review & editing, Writing – original draft, Supervision, Conceptualization. **Célia P.M. Bento:** Writing – review & editing, Investigation. **Carla S.S. Ferreira:** Writing – review & editing, Investigation. **António J.D. Ferreira:** Writing – review & editing, Resources, Methodology.

## Declaration of competing interest

The authors declare that they have no known competing financial interests or personal relationships that could have appeared to influence the work reported in this paper.
