# Supplementary material for: Occurrence and environmental risk assessment of pharmaceuticals in the Mondego river (Portugal)
Source: Heliyon. 2024 Jul 18;10(15):e34825. doi: 10.1016/j.heliyon.2024.e34825 (PMC11328081; doi:10.1016/j.heliyon.2024.e34825)
Supplement: Multimedia component 1 [file mmc1.docx]

# Appendix A. Supplementary

**Occurrence and environmental risk assessment of pharmaceuticals in the Mondego River (Portugal)**

*Danijela Kötke^1^*, Juergen Gandrass^1^, Célia P.M. Bento^1^, Carla S.S. Ferreira^2,3^, António J.D. Ferreira^2^*

^1^Helmholtz-Zentrum Hereon, [Institute of Coastal Environmental Chemistry](https://www.hereon.de/cms60/institutes/coastal_environmental_chemistry/index.php.en), Organic Environmental Chemistry, Geesthacht 21502, Germany

^2^Research Centre for Natural Resources, Environment and Society (CERNAS), Agrarian Technical School, Polytechnic Institute of Coimbra, P-3040-316, Coimbra, Portugal

^3^Department of Physical Geography and Bolin Centre for Climate Research, Stockholm University, SE-106 91 Stockholm, Sweden.

*Corresponding author: e-mail: danijela.koetke@hereon.de, Phone: +49-4152-87-2842; Fax: +49-4152-87-2332

**Table S1** Sampling parameters

| **River Mondego. Portugal** | | | | | | | |
| --- | --- | --- | --- | --- | --- | --- | --- |
| **September 2018 (04.09.2018 - 06.09.2018)** | | | | | | | |
| **Station** | **Date**  **UTC** | **Longitude**  **°E** | **Latitude**  **°N** | **Salinity**  **[µS cm^-1^]** | **pH** | **Temperature**  **[°C]** | **Water depth**  **[m]** |
| **M1** | 04.09.2018 11:04 | -8.267 | 40.281 | 80 | 6.9 | 19.0 | 1.5 |
| **M2** | 04.09.2018 11:41 | -8.280 | 40.266 | 76 | 7.1 | 20.0 | 1.0 |
| **M3** | 04.09.2018 13:40 | -8.280 | 40.260 | 75 | 7.2 | 20.0 | 1.0 |
| **M4** | 04.09.2018 14:55 | -8.289 | 40.240 | 82 | 7.3 | 21.2 | 1.0 |
| **M5** | 04.09.2018 15:35 | -8.302 | 40.239 | 79 | 7.4 | 20.9 | 0.5 |
| **M7** | 04.09.2018 17:14 | -8.366 | 40.202 | 77 | 7.5 | 21.7 | 1.0 |
| **M8** | 05.09.2018 09:32 | -8.394 | 40.185 | 77 | 7.8 | 19.7 | 0.2 |
| **M9** | 05.09.2018 10:11 | -8.402 | 40.182 | 78 | 7.6 | 19.6 | 0.3 |
| **M10** | 05.09.2018 11:11 | -8.428 | 40.200 | 100 | 7.7 | 21.4 | 2.0 |
| **M11** | 05.09.2018 12:05 | -8.454 | 40.220 | 100 | 7.9 | 23.4 | 0.4 |
| **M12** | 05.09.2018 15:45 | -8.548 | 40.199 | 167 | 7.7 | 21.7 | 1.0 |
| **M13** | 05.09.2018 16:17 | -8.616 | 40.183 | 147 | 8.0 | 24.2 | 0.5 |
| **M14** | 05.09.2018 17:07 | -8.671 | 40.163 | 211 | 7.7 | 22.9 | 0.4 |
| **M15** | 05.09.2018 17:34 | -8.676 | 40.152 | 188 | 7.9 | 23.2 | 0.1 |
| **M16** | 06.09.2018 15:02 | -8.713 | 40.144 | 964 | 7.9 | 23.9 | 0.2 |
| **M18** | 06.09.2018 16:12 | -8.817 | 40.142 | 36,900 | 8.6 | 23.2 | 0.3 |
| **M19** | 06.09.2018 17:00 | -8.867 | 40.146 | 47,000 | 8.4 | 19.4 | 1.0 |
| **A01** | 06.09.2018 18:00 | -8.864 | 40.129 | 36,000 | 8.5 | 19.5 | 0.3 |

**Table S2 LC-MS/MS parameters**

| **Liquid Chromatography** | | | | **Mass Spectrometry** | | |  |
| --- | --- | --- | --- | --- | --- | --- | --- |
| **Column** | Agilent ZORBAX Eclipse Plus C18 RRHD; 1.8 µm; 2.1x150 mm, (P.N. 959759-902) + Precolumn Agilent UHPLC Guard ZORBAX Eclipse Plus C18; 1.8 µm; 2.1x5 mm (P.N. 821725-901) | | | | **Ionisation mode** | Agilent Jet Stream Electrospray Ionisation (ESI) | |
| **Column temperature** | 20 °C | | | | **System** | Agilent 6490 Triple Quadrupole LC/MS System | |
| **System** | Agilent 1290 Infinity I LC System | | | | **Drying gas temperature** | 150 °C | |
| **Flow rate** | 0.2 mL min^-1^ | | | | **Drying gas flow** | 15 L min^-1^ | |
| **Injection volume** | 10 µL | | | | **Nebulizer pressure** | 30 psi | |
| **Mobile phase** | A: MilliQ-water + 0.1% formic acid | | | | **Sheath gas temperature** | 300 °C | |
|  | B: Methanol + 0.1% formic acid | | | | **Sheath gas flow** | 12 L min^-1^ | |
| **Gradient** | *Time (min)* | *%A* | *%B* | | **Capillary voltage** | 3500 V | |
|  | 4 | 85 | 15 | | **Nozzle voltage** | 0 V | |
|  | 13 | 65 | 35 | |  |  |  |
|  | 22 | 0 | 100 | |  |  |  |
|  | 32 | 0 | 100 | | **Polarity** | Positive | |
|  | 32.01 | 85 | 15 | | **Scan Type** | Dynamic MRM | |
| **Post time** | 2 min | | | | **Fragmentor voltage** | 380 V | |
| **Total run time** | 35 min | | | | **Cycle time** | 500 ms | |
|  |  | | | | **Ion Funnel Parameter:** |  | |
|  |  | | | | **High Pressure RF** | 150 V | |
|  |  | | | | **Low Pressure RF** | 60 V | |

**Table S3 Compound-specific LC-MS/MS parameters**

| **Class** | **Compound** | **Log K_OW_*** | **RT [min]** | **Precursor ion *[m/z]*** | **Quantifier *[m/z]* (CE [V])** | **Qualifier I *[m/z]* (CE [V])** | **Qualifier II *[m/z]* (CE [V])** | **CAV [V]** |
| --- | --- | --- | --- | --- | --- | --- | --- | --- |
| **Antibiotics** | Erythromycin | 2.48 | 20.2 | 734.5 | 158.1 (32) | 576.2 (20) | 83.0 (50) | 5 |
| ISTD | Erythromycin-13C, D3 |  | 20.2 | 738.5 | 162.1 (36) | 580.2 (20) | 82.9 (50) | 5 |
|  | Clarithromycin | 3.18 | 21.0 | 748.5 | 158.1 (28) | 590.2 (20) | 93.0 (50) | 5 |
|  | Roxithromycin | 2.75 | 21.1 | 837.5 | 679.2 (24) | 158.1 (40) | 116.1 (50) | 5 |
|  | Sulfamethoxazole | 0.48 | 15.4 | 254.1 | 65.0 (50) | 156.0 (16) | 108.0 (24) | 5 |
| ISTD | Sulfamethoxazole-13C6 |  | 15.4 | 260.1 | 70.0 (50) | 114.0 (28) | 162.0 (16) | 5 |
|  | Sulfamethazine | 0.76 | 12.2 | 279.1 | 108.0 (28) | 203.9 (16) | 124.0 (28) | 0 |
|  | Sulfadimethoxine | 1.17 | 18.4 | 311.1 | 156.0 (20) | 108.0 (32) | 65.0 (50) | 5 |
| ISTD | Sulfadimethoxine-D6 |  | 18.3 | 317.1 | 162.0 (28) | 155.9 (24) | 108.0 (36) | 5 |
|  | Tiamulin | 4.75 | 20.0 | 494.3 | 192.0 (20) | 163.1 (36) | 119.0 (48) | 5 |
|  | Lincomycin | 0.29 | 10.6 | 407.2 | 126.1 (32) | 359.1 (16) | 70.0 (50) | 5 |
| **ICM** | Iomeprol | -1.35 | 4.5 | 777.9 | 404.9 (48) | 686.6 (24) | 558.8 (32) | 5 |
| ISTD | Iomeprol-D3 |  | 4.4 | 780.9 | 407.9 (50) | 689.6 (24) | 561.8 (28) | 5 |
|  | Iopamidol | -1.38 | 3.1 | 777.9 | 558.7 (24) | 541.9 (36) | 386.8 (48) | 0 |
| ISTD | Iopamidol-D3 |  | 3.1 | 780.9 | 561.8 (24) | 544.8 (40) | 389.9 (50) | 0 |
|  | Iopromide | -2.49 | 8.2 | 791.9 | 572.8 (28) | 773.7 (20) | 558.8 (32) | 0 |
| ISTD | Iopromide-D3 |  | 8.0 | 794.9 | 575.8 (28) | 776.8 (24) | 561.8 (32) | 0 |
|  | Amidotrizoic acid | 1.37 | 4.4 | 614.8 | 361.0 (20) | 233.0 (50) | 192.0 (48) | 5 |
| ISTD | Amidotrizoic acid-D6 |  | 4.3 | 620.8 | 366.9 (24) | 239.0 (50) | 196.0 (50) | 5 |
|  | Ioxitalamic acid | 0.5 | 3.5 | 644.8 | 583.6 (16) | 301.8 (50) | 455.7 (32) | 0 |
|  | Iohexol | -2.81 | 4.3 | 821.9 | 803.8 (24) | 602.8 (32) | 375.0 (50) | 5 |
| **Analgesics** | Paracetamol | 0.27 | 7.4 | 152.1 | 110.0 (16) | 65.0 (36) | 43.1 (36) | 0 |
| ISTD | Paracetamol-D7 |  | 7.3 | 159.1 | 115.0 (20) | 69.0 (40) | 46.0 (36) | 0 |
|  | Diclofenac | 4.02 | 23.1 | 296.0 | 214.0 (40) | 277.8 (8) | 249.9 (12) | 5 |
| ISTD | Diclofenac-13C6 |  | 22.8 | 302.1 | 219.9 (32) | 255.8 (12) | 283.9 (4) | 5 |
| **Lipid reducer** | Bezafibrate | 4.25 | 22.2 | 362.1 | 138.9 (24) | 316.0 (12) | 121.1 (32) | 5 |
| ISTD | Bezafibrate-D6 |  | 22.1 | 368.2 | 138.9 (32) | 322.0 (16) | 121.0 (44) | 5 |
| **Antiepileptic** | Carbamazepine | 2.25 | 20.6 | 237.1 | 194.0 (20) | 179.0 (40) | 165.0 (50) | 5 |
| ISTD | Carbamazepine-D8 |  | 20.6 | 245.2 | 202.1 (24) | 199.0 (24) |  | 5 |
| **Anticonvulsant** | Primidone | 0.73 | 17.5 | 219.1 | 91.0 (30) | 162.0 (25) | 119.1 (20) | 5 |
| **Beta-Blocker** | Nadolol | 1.17 | 13.0 | 310.2 | 254.0 (16) | 201.0 (24) | 74.0 (28) | 5 |
|  | Propranolol | 2.6 | 19.0 | 260.2 | 116.1 (16) | 183.0 (10) | 74.0 (24) | 5 |
| ISTD | Propranolol-D7 |  | 19.0 | 267.2 | 123.1 (20) | 79.0 (20) | 57.0 (32) | 5 |
| **Injection-Standard** | Benzotriazole-D4 |  | 14.7 | 124.1 | 41.0 (44) | 96.1 (17) | 69.0 (28) | 5 |

*US EPA. 2016. Estimation Programs Interface Suite™for Microsoft® Windows, v.1.68. United States Environmentel Protection Agency, Washington, DC, USA. Estimated; ISTD: Internal Standard; RT: Retention time; CE: Collision Energy; CAV: Cell Accelerator Voltage

**Table S4 Overall method recoveries and %RSD**

| **Compound** | **Recovery [%] MilliQ water**  **spike 1.25 ng, pH 8,**  **1 L, *N*=3** | **Recovery [%] MilliQ water**  **spike 1.25 ng, pH 2,**  **1 L, *N*=3** | **Recovery [%] marine water,**  **spike 5 ng, pH 8, Sal.: 22.5, 1 L, *N*=3** | **Recovery [%] marine water,**  **spike 5 ng, pH 2, Sal.: 22.5, 1 L, *N*=3** | **Recovery [%] river water,**  **spike 7.5 ng, pH 8, Sal.: 3, 1 L, *N*=2** | **Recovery [%] river water,**  **spike 7.5 ng, pH 2, Sal.: 3, 1 L, *N*=2** |
| --- | --- | --- | --- | --- | --- | --- |
| Erythromycin | 67 ± 5 |  | 148 ± 13 |  | 75 - 80 |  |
| Clarithromycin | 56 ± 25 |  | 91 ± 5 |  | 69 - 90 |  |
| Roxithromycin | 92 ± 15 |  | 132 ± 16 |  | 111 - 115 |  |
| Sulfamethoxazole | 88 ± 14 |  | 106 ± 13 |  | 106 - 113 |  |
| Sulfamethazine | 60 ± 16 |  | 100 ± 5 |  | 101 - 107 |  |
| Sulfadimethoxine | 90 ± 15 |  | 123 ± 5 |  | 92 - 94 |  |
| Tiamulin | 88 ± 19 |  | 157 ± 10 |  | 33 - 37 |  |
| Lincomycin | 84 ± 45 |  | 115 ± 2 |  | 136 - 150 |  |
| Iomeprol | 79 ± 14 |  | 92 ± 10 |  | 98 - 134 |  |
| Iopamidol | 14 ± 2 |  | 68 ± 21 |  | 71 - 76 |  |
| Iopromide | 101 ± 18 |  | 85 ± 17 |  | 87 - 118 |  |
| Amidotrizoic acid |  | 97 ± 3 |  | 117 ± 33 |  | 76 - 118 |
| Ioxitalamic acid |  | 70 ± 12 |  | / |  | 43 - 46 |
| Iohexol | 70 ± 26 |  | 111 ± 13 |  | 64 - 81 |  |
| Paracetamol | 56 ± 5 |  | 115 ± 4 |  | 106 - 111 |  |
| Diclofenac | 65 ± 15 |  | 135 ± 8 |  | 63 - 109 |  |
| Bezafibrate | 73 ± 20 |  | 107 ± 4 |  | 62 - 68 |  |
| Carbamazepine | 88 ± 18 |  | 139 ± 8 |  | - |  |
| Primidone | 76 ± 5 |  | 70 ± 6 |  | 129 - 134 |  |
| Nadolol | 88 ± 26 |  | 107 ± 5 |  | 154 - 158 |  |
| Propranolol | 77 ± 9 |  | 106 ± 4 |  | 65 - 67 |  |

/: [unquantifiable](https://www.linguee.de/englisch-deutsch/uebersetzung/unquantifiable.html); RSD: Relative Standard Deviation

**Table S5 Instrumental and method detection and quantification limits**

| **Substance** | | **IDL [ng]** | | **IQL [ng]** | | **MDL_for surface water_ [ng L^-1^]** | | **MQL_for surface water_ [ng L^-1^]** | |
| --- | --- | --- | --- | --- | --- | --- | --- | --- | --- |
| **Sample Volume [L]** | | **1** | | **1** | | **1** | | **1** | |
| Erythromycin | 0.005 | | 0.01 | | 0.02 | | 0.06 | |  |
| Clarithromycin | 0.005 | | 0.01 | | <0.01 | | 0.01 | |  |
| Roxithromycin | 0.005 | | 0.01 | | 0.02 | | 0.07 | |  |
| Sulfamethoxazole | 0.005 | | 0.01 | | 0.02 | | 0.05 | |  |
| Sulfamethazine | 0.005 | | 0.01 | | 0.38 | | 1.28 | |  |
| Sulfadimethoxine | 0.005 | | 0.01 | | 0.01 | | 0.04 | |  |
| Tiamulin | 0.005 | | 0.01 | | 0.01 | | 0.02 | |  |
| Lincomycin | <0.001 | | 0.001 | | 0.01 | | 0.03 | |  |
| Iomeprol | 0.05 | | 0.10 | | 0.28 | | 0.47 | |  |
| Iopamidol | 0.05 | | 0.10 | | 0.33 | | 0.52 | |  |
| Iopromide | 0.05 | | 0.10 | | 0.37 | | 0.56 | |  |
| Amidotrizoic acid | 0.05 | | 0.10 | | 0.28 | | 0.65 | |  |
| Ioxitalamic acid | 0.05 | | 0.10 | | 2.26 | | 3.77 | |  |
| Iohexol | 0.03 | | 0.10 | | 0.76 | | 2.28 | |  |
| Paracetamol | 0.005 | | 0.01 | | 0.03 | | 0.10 | |  |
| Diclofenac | 0.02 | | 0.04 | | 0.08 | | 0.26 | |  |
| Bezafibrate | 0.05 | | 0.10 | | 0.03 | | 0.06 | |  |
| Carbamazepine | 0.005 | | 0.01 | | 0.05 | | 0.16 | |  |
| Primidone | 0.05 | | 0.10 | | 0.04 | | 0.07 | |  |
| Nadolol | 0.001 | | 0.005 | | 0.01 | | 0.04 | |  |
| Propranolol | 0.02 | | 0.05 | | 0.01 | | 0.03 | |  |

IDL: Instrumental detection limit; IQL: Instrumental quantification limit;

MDL: Method detection limit; MQL: Method quantification limit

**Table S6** Concentrations of Pharmaceuticals in the Mondego river [ng L^-1^] (September 2018)

| **Station** | **PCM** | **DCF** | **NAD** | **PRO** | **SMX** | **SMT** | **SDM** | **ERY** | **CLA** | **ROX** | **LIN** | **TIA** | **CBZ** | **PMD** | **BZF** | **IOP** | **IPM** | **IMI** | **IHX** | **DTZ** | **ITX** |
| --- | --- | --- | --- | --- | --- | --- | --- | --- | --- | --- | --- | --- | --- | --- | --- | --- | --- | --- | --- | --- | --- |
| **M1** | 0.17 | 3.08 | < 0.04* | 0.09 | 0.80 | n.d. | 0.09 | < 0.06* | 0.48 | < 0.07* | 0.25 | 0.07 | 6.96 | 1.64 | 0.10 | 7.19 | n.d. | 70.0 | n.d. | 4.06 | n.d. |
| **M2** | 0.28 | 2.35 | < 0.04* | 0.08 | 0.78 | n.d. | 0.08 | < 0.06* | 0.62 | < 0.07* | 0.24 | 0.05 | 6.75 | 1.51 | 0.09 | 7.99 | n.d. | 61.0 | n.d. | 2.97 | n.d. |
| **M3** | 0.18 | 2.04 | < 0.04* | 0.06 | 0.64 | n.d. | 0.07 | 0.06 | 0.48 | 0.12 | 0.25 | 0.10 | 6.07 | 1.48 | 0.07 | 8.17 | n.d. | 70.7 | n.d. | 2.75 | n.d. |
| **M4** | 0.16 | 2.91 | n.d. | 0.07 | 0.71 | n.d. | 0.07 | < 0.06* | 0.46 | 0.08 | 0.27 | 0.06 | 7.57 | 1.55 | 0.09 | 8.97 | n.d. | 70.9 | n.d. | 3.89 | n.d. |
| **M5** | 0.10 | 1.82 | n.d. | 0.07 | 0.44 | n.d. | 0.04 | < 0.06* | 0.45 | n.d. | 0.23 | 0.02 | 4.99 | 1.14 | 0.09 | 5.50 | n.d. | 43.1 | n.d. | 3.62 | n.d. |
| **M7** | < 0.10* | 1.35 | n.d. | 0.06 | 0.48 | n.d. | 0.04 | < 0.06* | 0.51 | n.d. | 0.25 | < 0.02* | 5.39 | 1.14 | < 0.06* | 5.24 | n.d. | 50.2 | n.d. | 2.99 | n.d. |
| **M8** | 0.21 | 2.06 | n.d. | 0.05 | 0.65 | n.d. | 0.06 | < 0.06* | 0.29 | n.d. | 0.26 | < 0.02* | 7.33 | 1.60 | < 0.06* | 6.67 | n.d. | 64.2 | n.d. | 3.42 | n.d. |
| **M9** | < 0.10* | 2.05 | n.d. | 0.05 | 0.69 | n.d. | 0.06 | < 0.06* | 0.35 | n.d. | 0.28 | < 0.02* | 6.62 | 1.54 | 0.07 | 7.04 | n.d. | 67.3 | n.d. | 3.32 | n.d. |
| **M10** | 0.48 | 1.19 | n.d. | 0.03 | 0.65 | n.d. | 0.05 | < 0.06* | 0.68 | n.d. | 0.32 | n.d. | 7.24 | 1.47 | < 0.06* | 6.56 | n.d. | 67.6 | n.d. | 3.58 | n.d. |
| **M11** | 0.27 | 0.99 | n.d. | n.d. | 0.67 | n.d. | 0.05 | < 0.06* | 0.46 | n.d. | 0.26 | n.d. | 6.48 | 1.68 | < 0.06* | 5.97 | n.d. | 50.2 | n.d. | 4.53 | n.d. |
| **M12** | 4.66 | 59.8 | n.d. | 1.47 | 12.9 | n.d. | 0.05 | 3.76 | 4.65 | 0.20 | 0.70 | 0.06 | 52.6 | 13.5 | 10.2 | 386 | 4.31 | 2810 | 70.5 | 37.7 | n.d. |
| **M13** | 2.79 | 11.7 | n.d. | 0.47 | 5.68 | n.d. | 0.05 | 1.51 | 2.62 | n.d. | 0.54 | n.d. | 27.7 | 7.11 | 3.95 | 104 | n.d. | 1230 | 10.1 | 27.7 | n.d. |
| **M14** | 1.88 | 17.6 | n.d. | 0.48 | 7.49 | n.d. | 0.05 | 0.42 | 1.46 | n.d. | 0.60 | n.d. | 42.2 | 8.16 | 3.94 | 156 | n.d. | 1010 | < 2.28* | 26.5 | n.d. |
| **M15** | 2.61 | 13.7 | n.d. | 0.37 | 7.58 | n.d. | 0.06 | 0.31 | 1.54 | n.d. | 0.59 | n.d. | 36.5 | 7.27 | 3.23 | 149 | n.d. | 895 | < 2.28* | 18.5 | n.d. |
| **M16** | 1.81 | 5.11 | n.d. | 0.25 | 7.08 | n.d. | 0.05 | 0.26 | 1.69 | n.d. | 0.46 | < 0.02* | 23.2 | 4.78 | 1.35 | 129 | n.d. | 601 | < 2.28* | 5.0 | n.d. |
| **M18** | 2.35 | 14.4 | n.d. | 1.88 | 7.17 | n.d. | 0.04 | 0.21 | 3.04 | n.d. | 0.57 | < 0.02* | 26.6 | 6.51 | 0.64 | 38.8 | n.d. | 218 | < 2.28* | 0.82 | n.d. |
| **M19** | 14.2 | 0.53 | n.d. | 0.07 | 1.26 | n.d. | n.d. | n.d. | 0.24 | n.d. | 0.06 | n.d. | 2.28 | 0.56 | 0.37 | 6.55 | n.d. | 44.8 | n.d. | n.d. | n.d. |
| **A01** | 1.61 | n.d. | n.d. | n.d. | 0.09 | n.d. | n.d. | n.d. | 0.01 | n.d. | 0.03 | n.d. | 0.22 | n.d. | n.d. | 0.63 | n.d. | 3.03 | n.d. | n.d. | n.d. |

*< MQL: smaller than method quantification limit; n.d.: not detected; PCM: Paracetamol; DCF: Diclofenac; NAD: Nadolol; PRO: Propranolol; SMX: Sulfamethoxazole;

SMT: Sulfamethazine; SDM: Sulfadimethoxine; ERY: Erythromycin; CLA: Clarithromycin; ROX: Roxithromycin; LIN: Lincomycin; TIA: Tiamulin; CBZ: Carbamazepine;

PMD: Primidone; BZF: Bezafibrate; IOP: Iomeprol; IPM: Iopamidol; IMI: Iopromide; IHX: Iohexol; DTZ: Amidotricoic acid; ITX: Ioxitalamic acid

**Table S7** Occurrence of pharmaceuticals in aquatic environments. Concentrations in [ng L^-1^].

| **Sampling location** | **Erythromycin** | **Clarithromycin** | **Sulfamethoxazole** | **Carbamazepine** | **Bezafibrate** | **Iomeprol** | **Iopromide** | **Amidotricoic acid** | **Diclofenac** | **Paracetamol** | **References** |
| --- | --- | --- | --- | --- | --- | --- | --- | --- | --- | --- | --- |
| Mondego River. P. Sept 2018 | < MQL - 3.76^2)^ | 0.01 - 4.65^2)^ | 0.09 - 12.9^2)^ | 0.22 - 52.6^2)^ | n.d. - 10.2^2)^ | 0.63 - 386^2)^ | 3.03 - 2810^2)^ | n.d. - 37.7^2)^ | n.d. - 17.6^2)^ | 0.10 - 14.2^2)^ | This study |
| Douro River Estuary. P | n.a. | n.a. | 53.3^4)^ | 178^4)^ | n.a. | n.a. | n.a. | n.a. | n.a. | n.a. | ([Madureira et al., 2010](#_ENREF_38)) |
| Llobregat River / Anoia. E | 10 - 70^2)^ | n.a. | 30 - 11,920^2)^ | 80 - 3,090^2)^ | 30 - 15,060^2)^ | n.a. | n.a. | n.a. | 0.08 - 18.74^2)^ | 60 - 2,420^2)^ | ([Ginebreda et al., 2010](#_ENREF_23)) |
| SW. Ebro. E | n.d. | LOQ - 37^2)^ | LOD - 50^2)^ | 1 - 60^2)^ | 0.3 - 25^2)^ | n.a. | n.a. | n.a. | LOQ - 219^2)^ | n.d. - 39^2)^ | ([Gros et al., 2009](#_ENREF_24)) |
| Rivers. D. May-August 2015 | 0.17 - 3.90^2)^ | 0.13 - 8.07^2)^ | 3.35 - 42.6^2)^ | 57.6 - 162^2)^ | n.d. - 6.02^2)^ | 21.1 - 488^2)^ | 21.0 - 123^2)^ | 61.5 - 222^2)^ | n.d. - 27.1^2)^ | n.d. | ([Kotke et al., 2019](#_ENREF_30)) |
| Rhine River. Germany. D | n.d. | 1.0 - 4.1^2)^ | 1.9 - 5.0^2)^ | 9.5 - 23^2)^ | 0.8 - 1.0 | 60 - 160^2) including Iopamidol^ | 25 - 89^2)^ | n.d. | 5.4 - 17^2)^ | n.d. - 29^2)^ | (Singer et al., 2009) |
| Rhine (Constance-Lobith). D | n.a. | 2 - 20 | 8 - 45 | 10 - 85 | n.a. | n.a. | n.a. | n.a. | 2 - 75 | n.a. | ([Ruff et al., 2015](#_ENREF_55)) |
| Jialing River. China | 12 - 23^2)^ | n.a. | 18 - 21^2)^ | n.a. | n.a. | n.a. | n.a. | n.a. | n.a. | n.a. | ([Chang et al., 2010](#_ENREF_11)) |
| Atlantic Ocean. coast. P | n.a. | n.a. | n.a. | n.a. | n.a. | n.a. | n.a. | n.a. | n.d. - 241^2)^ | 51.2 - 584^2)^ | ([Lolic et al., 2015](#_ENREF_36)) |
| Coastal wetland Valencia. E | n.a. | n.a. | n.a. | n.a. | n.d. - 79^2)^ | n.a. | n.a. | n.a. | n.d. - 169^2)^ | n.d. - 168^2)^ | ([Sadutto et al., 2021](#_ENREF_56)) |
| Coastal water Cádiz. E  Oceanic water Cádiz. E | n.d. - 2.3^2)^  < LOD - 0.3^2)^ | 0.2 - 9.4^2)^  n.d. | n.d. - 99^2)^  n.d. | n.d. - 31.1^2)^  n.d. - 0.1^2)^ | n.d. - 0.5^2)^  n.d. | n.a. | n.a. | n.a. | n.d. - 31.9^2)^  n.d. - 2.5^2)^ | n.d. - 41.5^2)^  n.d. - 2.8^2)^ | ([Biel-Maeso et al., 2018](#_ENREF_6)) |
| Northern Adriatic Sea. I | 5.8^1)^ | n.d. | 3.6^1)^ | 3.1^1)^ | n.a. | 29^1)^ | n.d. | n.a. | n.d. | n.d. | ([Nodler et al., 2014](#_ENREF_42)) |
| Northern Adriatic Sea. I | n.a. | n.a. | 0.265 - 1.02^2)^ | 0.11 - 0.36^2)^ | 0.02 - 0.14^2)^ | n.a. | n.a. | n.a. | n.a. | n.a. | ([Loos et al., 2013](#_ENREF_37)) |
| Aegean Sea&Dardanelles. GR/TR | n.d. | 16^1)^ | 3.8^1)^ | 2.9^1)^ | 3.5^1)^ | 83^1)^ | 109^1)^ | n.a. | 4.6^1)^ | 39^1)^ | ([Nodler et al., 2014](#_ENREF_42)) |
| Baltic Sea. D. 2015-2016 | n.d. - 0.14^2)^ | 0.03 - 0.42^2)^ | 0.74 - 3.29^2)^ | 1.98 - 10.6^2)^ | n.d. - 0.64^2)^ | 1.05 - 34.5^2)^ | 0.42 - 3.34^2)^ | n.a. | n.d. - 0.84^2)^ | 0.42 - 0.57^2)^ | ([Kotke et al., 2019](#_ENREF_30)) |
| German Bight. D. May 2015 | 0.13 - 0.94^2)^ | 0.4 - 1.66^2)^ | 1.78 - 13.0^2)^ | 4.78 - 29.7^2)^ | < MQL - 2.06^2)^ | 7.66 - 207^2)^ | 7.27 - 34.1^2)^ | 1.71 - 86.9^2)^ | < MQL - 4.82^2)^ | n.d. | ([Kotke et al., 2019](#_ENREF_30)) |
| Baltic Sea. D | n.d. | 14^1)^ | 21^1)^ | 22^1)^ | n.d. | 98^1)^ | 45^1)^ | n.a. | 9.2^1)^ | 48^1)^ | ([Nodler et al., 2014](#_ENREF_42)) |
| North Sea. B | n.a. | n.a. | n.d. | n.d. - 19^2)^ | n.d. - 8^2)^ | n.a. | n.a. | n.a. | n.a. | n.a. | (Wille et al.. 2010) |
| Mondego WWTP effluent. P | 20.4 - 134^2)^ | 12.0 - 40.0^2)^ | 340 - 1,679^2)^ | 364 - 496^2)^ | 93.8 - 635^2)^ | n.a. | 33,885 - 85,000^2)^ | n.a. | 24.6 - 83.1^2)^ | 83.1 - 106^2)^ | ([Santos et al., 2013](#_ENREF_58)) |
| WWTP effluent. Mondego. P  Spring  Summer  Autumn  Winter | n.a | n.a. | n.a. | n.a. | 25.1 - 58.4^2)^  560 - 590^2)^  150 - 4,200^2)^  1,100 - 8,200^2)^ | n.a. | n.a. | n.a. | 73.0 - 78.5^2)^  n.d.  n.d.  n.d. | n.d. - 530.7^2)^  n.d.  n.d. - 14,700^2)^  n.d. - 24,000^2)^ | ([Pereira et al., 2016](#_ENREF_48)) |

n.d.: not detected; n.a.: not analysed; < MQL: smaller than method quantification limit; LOD: Limit of detection; LOQ: Limit of Quantification; ^1)^ mean concentration; ^2)^ minimum-maximum concentration; ^3)^median concentration; ^4)^maximum concentration; B: Belgium; CH: Switzerland; D: Germany; E: Spain; GR: Greece; I: Italy; IRL: Ireland; P: Portugal; PL: Poland;

S: Sweden; TR: Turkey; US: United States; YS: Yellow Sea

**Table S8** Environmental risk assessment – freshwater at station M1-M11 (< 1 psu)

| **Compound** | **Organism** | | | **Endpoint** | | | **Duration** | **Effect conc. [µg L^-1^]** | | **Assessment factor**^1)^ | **PNEC^1)^ [µg L^-1^]** | **MECmax [µg L^-1^]** | | | **RQmax** | |
| --- | --- | --- | --- | --- | --- | --- | --- | --- | --- | --- | --- | --- | --- | --- | --- | --- |
| Erythromycin | *Selenastrum capricornutum* | | | growth | | |  | 1.03E+01 | NOEC^2)^ | 50 | 2.06E-01 | 5.80E-05 | | | 2.82E-04 | |
|  | *Anabaena cylindrica-NIES 19* | | | growth | | | 144h | 3.10E+00 | NOEC^3)^ | 50 | 6.20E-02 |  | | | 9.35E-04 | |
|  | *Oncorhynchus mykiss* | | | oxidative stress (gills) | | | 28d | 4.00E-01 | EC^4)^ | 50 | 8.00E-03 |  | | | 7.25E-03 | |
|  | *Oncorhynchus mykiss* | | | oxidative stress (gills) | | | 28d | 2.00E-01 | EC^4)^ | 50 | 4.00E-03 |  | | | 1.45E-02 | |
| Clarithromycin | *Pseudokirchneriella subcapitata* | | | growth | | | 72h | 2.00E+00 | EC50^5)^ | 10 | 2.00E-01 | 6.82E-04 | | | 3.41E-03 | |
| Roxithromycin | *Lemna gibba* | | | arotinoid | | | 9d | 1.00E+03 | EC50^6)^ | 5000 | 2.00E-01 | 1.23E-04 | | | 6.16E-04 | |
| Sulfamethoxazole | *Synechococcus leopoliensis* | | | growth | | | 96h | 5.90E+00 | NOEC^7)^ | 10 | 5.90E-01 | 8.01E-04 | | | 1.36E-03 | |
| Sulfadimethoxine | *Lemna minor* | | | growth | | |  | 2.00E+01 | EC50^8)^ | 5000 | 4.00E-03 | 9.49E-05 | | | 2.37E-02 | |
| Tiamulin | *Microcystis aeruginosa* | | | growth | | | 7d | 3.00E+00 | EC50^9)^ | 1000 | 3.00E-03 | 9.81E-05 | | | 3.27E-02 | |
| Lincomycin | *Pseudokirchneriella subcapitata* | | | growth | | | 72h | 7.00E+01 | LC50^5)^ | 1000 | 7.00E-02 | 3.25E-04 | | | 4.64E-03 | |
| Iomeprol |  | | |  | | |  |  |  |  |  | 8.97E-03 | | |  | |
| Iopamidol |  | | |  | | |  |  |  |  |  |  | | |  | |
| Iopromide |  | | |  | | |  | 6.80E+04 | ^1)^ | 10 | 6.80E+03 | 7.09E-02 | | | 1.04E-05 | |
| Amidotrizoic acid | *Daphnia magna* | | | mobility | | | 48h | 1.00E+05 | NOEC^10)^ | 10 | 1.00E+04 | 4.53E-03 | | | 4.53E-07 | |
| Iohexol | *Daphnia magna* | | | mobility | | | 48h | 1.00E+05 | NOEC^10)^ | 100 | 1.00E+03 | 0.00E+00 | | | 0.00E+00 | |
| Paracetamol | *Pimephales promelas* | | | reproduction | | |  | 1.00E+03 | EC50^11)^ | 1000 | 1.00E+00 | 4.78E-04 | | | 4.78E-04 | |
| Diclofenac | *Oncorhynchus mykiss* | | | cytopathology (liver) | | | 28d | 1.00E+00 | LOEC^12)^ | 10 | 1.00E-01 | 3.08E-03 | | | 3.08E-02 | |
|  | *Salmo trout f. fario* | | | histopathohogical | | | 21d | 5.00E-01 | NOEC^13)^ | 10 | 5.00E-02 |  | | | 6.17E-02 | |
| Bezafibrate | *Pimephales promelas* | | | repoduction | | |  | 5.30E+03 | EC50^11)^ | 5000 | 1.06E+00 | 1.02E-04 | | | 9.58E-05 | |
|  | *Ceriodaphnia dubia* | | | growth inhibition | | | 7d | 2.30E+01 | NOEC^14)^ | 5000 | 4.60E-03 |  | | | 2.21E-02 | |
| **Carbamazepine** | *Ceriodaphnia dubia* | | | reproduction | | | 7d | 2.50E+01 | NOEC^7)^ | 10 | 2.50E+00 | 7.57E-03 | | | 3.03E-03 | |
|  | *Cyprinus carpio* | | | cytopathology (kidney) | | | 28d | 1.00E+00 | LOEC^12)^ | 10 | 1.00E-01 |  | | | 7.57E-02 | |
|  | *Gammarus pulex* | | | behaviour | | |  | 1.00E-02 | LOEC^15)^ | 10 | 1.00E-03 |  | | | **7.57E+00** | |
| Primidone |  | | |  | | |  | 1.60E+01 | ^1)^ | 50 | 3.20E-01 | 1.68E-03 | | | 5.25E-03 | |
| Nadolol |  | | |  | | |  | 1.00E+02 | ^1)^ | 5000 | 2.00E-02 |  | | |  | |
| Propranolol | *Hyalella azteca* | | | reproduction | | | 7d | 1.00E+00 | NOEC^16)^ | 10 | 1.00E-01 | 8.66E-05 | | | 8.66E-04 | |
| *0.1 ≤ RQ < 1: medium risk*; | | **RQ ≥ 1: high risk** |  | |  |  | | | | |  |  |  |  | |  |
| ^1)^(Bergmann et al. 2011); ^2)^([Eguchi et al. 2004](#_ENREF_25)); ^3)^([Ando et al. 2007](#_ENREF_3)); ^4)^([Rodrigues et al. 2016](#_ENREF_71)); ^5)^[(Isidori et al. 2005](#_ENREF_41)); ^6)^([Brain et al. 2004](#_ENREF_12)); ^7)^([Ferrari et al. 2004](#_ENREF_29)); ^8)^([Bialk-Bielinska et al. 2011](#_ENREF_9)); ^9)^(Hallig-Soerensen et al. 2000); ^10)^(Steger-Hartmann et al. 1998); ^11)^([Sanderson et al. 2003](#_ENREF_76)); ^12)^[(Triebskorn et al. 2007](#_ENREF_81)); ^13)^[(Hoeger et al. 2005](#_ENREF_38)); ^14)^[(Isidori et al. 2007](#_ENREF_42)); ^15)^([De Lange et al. 2006](#_ENREF_15)); ^16)^([Huggett et al. 2002](#_ENREF_40)). | | | | | | | | | | | | | | | | |

**Table S9** Environmental risk assessment – freshwater at station M12 (< 1 psu)

| **Compound** | **Organism** | | | **Endpoint** | | | **Duration** | **Effect conc. [µg L^-1^]** | | **Assessment factor**^1)^ | **PNEC^1)^ [µg L^-1^]** | **MECmax [µg L^-1^]** | | | **RQmax** | |
| --- | --- | --- | --- | --- | --- | --- | --- | --- | --- | --- | --- | --- | --- | --- | --- | --- |
| *Erythromycin* | *Selenastrum capricornutum* | | | growth | | |  | 1.03E+01 | NOEC^2)^ | 50 | 2.06E-01 | 3.76E-03 | | | 1.83E-02 | |
|  | *Anabaena cylindrica-NIES 19* | | | growth | | | 144h | 3.10E+00 | NOEC^3)^ | 50 | 6.20E-02 |  | | | 6.06E-02 | |
|  | *Oncorhynchus mykiss* | | | oxidative stress (gills) | | | 28d | 4.00E-01 | EC^4)^ | 50 | 8.00E-03 |  | | | *4.70E-01* | |
|  | *Oncorhynchus mykiss* | | | oxidative stress (gills) | | | 28d | 2.00E-01 | EC^4)^ | 50 | 4.00E-03 |  | | | *9.40E-01* | |
| Clarithromycin | *Pseudokirchneriella subcapitata* | | | growth | | | 72h | 2.00E+00 | EC50^5)^ | 10 | 2.00E-01 | 4.65E-03 | | | 2.33E-02 | |
| Roxithromycin | *Lemna gibba* | | | arotinoid | | | 9d | 1.00E+03 | EC50^6)^ | 5000 | 2.00E-01 | 2.03E-04 | | | 1.02E-03 | |
| Sulfamethoxazole | *Synechococcus leopoliensis* | | | growth | | | 96h | 5.90E+00 | NOEC^7)^ | 10 | 5.90E-01 | 1.29E-02 | | | 2.18E-02 | |
| Sulfadimethoxine | *Lemna minor* | | | growth | | |  | 2.00E+01 | EC50^8)^ | 5000 | 4.00E-03 | 5.39E-05 | | | 1.35E-02 | |
| Tiamulin | *Microcystis aeruginosa* | | | growth | | | 7d | 3.00E+00 | EC50^9)^ | 1000 | 3.00E-03 | 5.79E-05 | | | 1.93E-02 | |
| Lincomycin | *Pseudokirchneriella subcapitata* | | | growth | | | 72h | 7.00E+01 | LC50^5)^ | 1000 | 7.00E-02 | 7.01E-04 | | | 1.00E-02 | |
| Iomeprol |  | | |  | | |  |  |  |  |  | 3.86E-01 | | |  | |
| Iopamidol |  | | |  | | |  |  |  |  |  | 4.31E-03 | | |  | |
| Iopromide |  | | |  | | |  | 6.80E+04 | ^1)^ | 10 | 6.80E+03 | 2.81E+00 | | | 4.13E-04 | |
| Amidotrizoic acid | *Daphnia magna* | | | mobility | | | 48h | 1.00E+05 | NOEC^10)^ | 10 | 1.00E+04 | 3.77E-02 | | | 3.77E-06 | |
| Iohexol | *Daphnia magna* | | | mobility | | | 48h | 1.00E+05 | NOEC^10)^ | 100 | 1.00E+03 | 7.05E-02 | | | 7.05E-05 | |
| Paracetamol | *Pimephales promelas* | | | reproduction | | |  | 1.00E+03 | EC50^11)^ | 1000 | 1.00E+00 | 4.66E-03 | | | 4.66E-03 | |
| **Diclofenac** | *Oncorhynchus mykiss* | | | cytopathology (liver) | | | 28d | 1.00E+00 | LOEC^12)^ | 10 | 1.00E-01 | 5.98E-02 | | | *5.98E-01* | |
|  | *Salmo trout f. fario* | | | histopathohogical | | | 21d | 5.00E-01 | NOEC^13)^ | 10 | 5.00E-02 |  | | | **1.20E+00** | |
| **Bezafibrate** | *Pimephales promelas* | | | repoduction | | |  | 5.30E+03 | EC50^11)^ | 5000 | 1.06E+00 | 1.02E-02 | | | 9.63E-03 | |
|  | *Ceriodaphnia dubia* | | | growth inhibition | | | 7d | 2.30E+01 | NOEC^14)^ | 5000 | 4.60E-03 |  | | | **2.22E+00** | |
| **Carbamazepine** | *Ceriodaphnia dubia* | | | reproduction | | | 7d | 2.50E+01 | NOEC^7)^ | 10 | 2.50E+00 | 5.26E-02 | | | 2.11E-02 | |
|  | *Cyprinus carpio* | | | cytopathology (kidney) | | | 28d | 1.00E+00 | LOEC^12)^ | 10 | 1.00E-01 |  | | | *5.26E-01* | |
|  | *Gammarus pulex* | | | behaviour | | |  | 1.00E-02 | LOEC^15)^ | 10 | 1.00E-03 |  | | | **5.26E+01** | |
| Primidone |  | | |  | | |  | 1.60E+01 | ^1)^ | 50 | 3.20E-01 | 1.35E-02 | | | 4.22E-02 | |
| Nadolol |  | | |  | | |  | 1.00E+02 | ^1)^ | 5000 | 2.00E-02 |  | | |  | |
| Propranolol | *Hyalella azteca* | | | reproduction | | | 7d | 1.00E+00 | NOEC^16)^ | 10 | 1.00E-01 | 1.47E-03 | | | 1.47E-02 | |
| *0.1 ≤ RQ < 1: medium risk*; | | **RQ ≥ 1: high risk** |  | |  |  | | | | |  |  |  |  | |  |
| ^1)^(Bergmann et al. 2011); ^2)^([Eguchi et al. 2004](#_ENREF_25)); ^3)^([Ando et al. 2007](#_ENREF_3)); ^4)^([Rodrigues et al. 2016](#_ENREF_71)); ^5)^[(Isidori et al. 2005](#_ENREF_41)); ^6)^([Brain et al. 2004](#_ENREF_12)); ^7)^([Ferrari et al. 2004](#_ENREF_29)); ^8)^([Bialk-Bielinska et al. 2011](#_ENREF_9)); ^9)^(Hallig-Soerensen et al. 2000); ^10)^(Steger-Hartmann et al. 1998); ^11)^([Sanderson et al. 2003](#_ENREF_76)); ^12)^[(Triebskorn et al. 2007](#_ENREF_81)); ^13)^[(Hoeger et al. 2005](#_ENREF_38)); ^14)^[(Isidori et al. 2007](#_ENREF_42)); ^15)^([De Lange et al. 2006](#_ENREF_15)); ^16)^([Huggett et al. 2002](#_ENREF_40)). | | | | | | | | | | | | | | | | |

**Table S10** Environmental risk assessment – freshwater at station M13 - M16 (< 1 psu)

| **Compound** | **Organism** | | | **Endpoint** | | | **Duration** | **Effect conc. [µg L^-1^]** | | **Assessment factor**^1)^ | **PNEC^1)^ [µg L^-1^]** | **MECmax [µg L^-1^]** | | | **RQmax** | |
| --- | --- | --- | --- | --- | --- | --- | --- | --- | --- | --- | --- | --- | --- | --- | --- | --- |
| *Erythromycin* | *Selenastrum capricornutum* | | | growth | | |  | 1.03E+01 | NOEC^2)^ | 50 | 2.06E-01 | 1.51E-03 | | | 7.32E-03 | |
|  | *Anabaena cylindrica-NIES 19* | | | growth | | | 144h | 3.10E+00 | NOEC^3)^ | 50 | 6.20E-02 |  | | | 2.43E-02 | |
|  | *Oncorhynchus mykiss* | | | oxidative stress (gills) | | | 28d | 4.00E-01 | EC^4)^ | 50 | 8.00E-03 |  | | | *1.88E-01* | |
|  | *Oncorhynchus mykiss* | | | oxidative stress (gills) | | | 28d | 2.00E-01 | EC^4)^ | 50 | 4.00E-03 |  | | | *3.77E-01* | |
| Clarithromycin | *Pseudokirchneriella subcapitata* | | | growth | | | 72h | 2.00E+00 | EC50^5)^ | 10 | 2.00E-01 | 2.62E-03 | | | 1.31E-02 | |
| Roxithromycin | *Lemna gibba* | | | arotinoid | | | 9d | 1.00E+03 | EC50^6)^ | 5000 | 2.00E-01 | 0.00E+00 | | | 0.00E+00 | |
| Sulfamethoxazole | *Synechococcus leopoliensis* | | | growth | | | 96h | 5.90E+00 | NOEC^7)^ | 10 | 5.90E-01 | 7.58E-03 | | | 1.28E-02 | |
| Sulfadimethoxine | *Lemna minor* | | | growth | | |  | 2.00E+01 | EC50^8)^ | 5000 | 4.00E-03 | 5.65E-05 | | | 1.41E-02 | |
| Tiamulin | *Microcystis aeruginosa* | | | growth | | | 7d | 3.00E+00 | EC50^9)^ | 1000 | 3.00E-03 | 0.00E+00 | | | 0.00E+00 | |
| Lincomycin | *Pseudokirchneriella subcapitata* | | | growth | | | 72h | 7.00E+01 | LC50^5)^ | 1000 | 7.00E-02 | 6.04E-04 | | | 8.63E-03 | |
| Iomeprol |  | | |  | | |  |  |  |  |  | 1.56E-01 | | |  | |
| Iopamidol |  | | |  | | |  |  |  |  |  | 0.00E+00 | | |  | |
| Iopromide |  | | |  | | |  | 6.80E+04 | ^1)^ | 10 | 6.80E+03 | 1.23E+00 | | | 1.81E-04 | |
| Amidotrizoic acid | *Daphnia magna* | | | mobility | | | 48h | 1.00E+05 | NOEC^10)^ | 10 | 1.00E+04 | 2.77E-02 | | | 2.77E-06 | |
| Iohexol | *Daphnia magna* | | | mobility | | | 48h | 1.00E+05 | NOEC^10)^ | 100 | 1.00E+03 | 1.01E-02 | | | 1.01E-05 | |
| Paracetamol | *Pimephales promelas* | | | reproduction | | |  | 1.00E+03 | EC50^11)^ | 1000 | 1.00E+00 | 2.79E-03 | | | 2.79E-03 | |
| *Diclofenac* | *Oncorhynchus mykiss* | | | cytopathology (liver) | | | 28d | 1.00E+00 | LOEC^12)^ | 10 | 1.00E-01 | 1.76E-02 | | | *1.76E-01* | |
|  | *Salmo trout f. fario* | | | histopathohogical | | | 21d | 5.00E-01 | NOEC^13)^ | 10 | 5.00E-02 |  | | | *3.52E-01* | |
| *Bezafibrate* | *Pimephales promelas* | | | repoduction | | |  | 5.30E+03 | EC50^11)^ | 5000 | 1.06E+00 | 3.95E-03 | | | 3.73E-03 | |
|  | *Ceriodaphnia dubia* | | | growth inhibition | | | 7d | 2.30E+01 | NOEC^14)^ | 5000 | 4.60E-03 |  | | | *8.58E-01* | |
| **Carbamazepine** | *Ceriodaphnia dubia* | | | reproduction | | | 7d | 2.50E+01 | NOEC^7)^ | 10 | 2.50E+00 | 4.22E-02 | | | 1.69E-02 | |
|  | *Cyprinus carpio* | | | cytopathology (kidney) | | | 28d | 1.00E+00 | LOEC^12)^ | 10 | 1.00E-01 |  | | | *4.22E-01* | |
|  | *Gammarus pulex* | | | behaviour | | |  | 1.00E-02 | LOEC^15)^ | 10 | 1.00E-03 |  | | | **4.22E+01** | |
| Primidone |  | | |  | | |  | 1.60E+01 | ^1)^ | 50 | 3.20E-01 | 8.16E-03 | | | 2.55E-02 | |
| Nadolol |  | | |  | | |  | 1.00E+02 | ^1)^ | 5000 | 2.00E-02 |  | | |  | |
| Propranolol | *Hyalella azteca* | | | reproduction | | | 7d | 1.00E+00 | NOEC^16)^ | 10 | 1.00E-01 | 4.78E-04 | | | 4.78E-03 | |
| *0.1 ≤ RQ < 1: medium risk*; | | **RQ ≥ 1: high risk** |  | |  |  | | | | |  |  |  |  | |  |
| ^1)^(Bergmann et al. 2011); ^2)^([Eguchi et al. 2004](#_ENREF_25)); ^3)^([Ando et al. 2007](#_ENREF_3)); ^4)^([Rodrigues et al. 2016](#_ENREF_71)); ^5)^[(Isidori et al. 2005](#_ENREF_41)); ^6)^([Brain et al. 2004](#_ENREF_12)); ^7)^([Ferrari et al. 2004](#_ENREF_29)); ^8)^([Bialk-Bielinska et al. 2011](#_ENREF_9)); ^9)^(Hallig-Soerensen et al. 2000); ^10)^(Steger-Hartmann et al. 1998); ^11)^([Sanderson et al. 2003](#_ENREF_76)); ^12)^[(Triebskorn et al. 2007](#_ENREF_81)); ^13)^[(Hoeger et al. 2005](#_ENREF_38)); ^14)^[(Isidori et al. 2007](#_ENREF_42)); ^15)^([De Lange et al. 2006](#_ENREF_15)); ^16)^([Huggett et al. 2002](#_ENREF_40)). | | | | | | | | | | | | | | | | |

**Table S11** Environmental risk assessment - brackish (1-10 psu) and marine (> 10 psu) water samples at stations M18 - A01

| **Compound** | **Organisms** | | **Endpoint** | | **Duration** | | **Effect conc. [µg L^-1^]** | | | **Assessment factor** | | **PNEC [µg L^-1^]** | **MECmax [µg L^-1^]** | | **RQmax** |
| --- | --- | --- | --- | --- | --- | --- | --- | --- | --- | --- | --- | --- | --- | --- | --- |
| Erythromycin | *Chlorella vulgaris* | | growth | | 96h | | 8.57E+01 | (EC50)^1)^ | | 1.00E+03 | | 8.57E-02 | 2.07E-04 | | 2.41E-03 |
|  | *Synechococcus sp. PCC 7002* | | growth | | 144h | | 7.80E+00 | (NOEC)^2)^ | |  | | 7,80E-03 |  | | 2,65E-02 |
| **Clarithromycin** | *Skeletonema marinoi* | | growth | | 72h | | 1.52E-01 | (EC50)^3)^ | | 1.00E+03 | | 1.52E-04 | 3.04E-03 | | **2.00E+01** |
| Sulfamethoxazole | *Skeletonema marinoi* | | growth | | 72h | | 5.35E+03 | (EC50)^3)^ | | 1.00E+04 | | 5.35E-01 | 7.17E-03 | | 1.34E-02 |
|  | *Cyclotella meneghiniana* | | growth | | 96h | | 1.25E+03 | (NOEC)^4)^ | |  | | 1.25E-01 |  | | 5.74E-02 |
| Sulfadimethoxine | *Artemia nauplii* | | lethality | | 96h | | 1.95E+04 | (LC50)^5)^ | | 1.00E+04 | | 1.95E+00 | 3.96E-05 | | 2.03E-05 |
| Lincomycin | *Cyclotella meneghiniana* | | growth | | 96h | | 7.81E+02 | (NOEC)^6)^ | | 1.00E+04 | | 7.81E-02 | 5.73E-04 | | 7.33E-03 |
| Iopromide | *Leuciscus idus melanotus* | | mortality | | 48h | | 1.00E+07 | (LC50)^7)^ | | 1.00E+04 | | 1.00E+03 | 2.18E-01 | | 2.18E-04 |
|  | *Vibrio fischeri* | | luminescence | | 30min | | 1.00E+07 | (EC50)^8)^ | |  | | 1.00E+03 |  | | 2.18E-04 |
|  | *Lepomis macrochirus* | | mortality | |  | | 9.73E+05 | (LC50)^9)^ | |  | | 9.73E+01 |  | | 2.24E-03 |
| Paracetamol | *Artemia salina* | | immobilisation | | 48h | | 1.00E+05 | (EC50)^3)^ | | 1.00E+04 | | 1.00E+01 | 1.42E-02 | | 1.42E-03 |
|  | *Photobacterium phosphoreum* | | luminescence | | 15min | | 5.55E+04 | (L)EC50^10)^ | |  | | 5.55E+00 |  | | 2.55E-03 |
| Diclofenac | *Vibrio fischeri* | | luminescence | | 30min | | 1.15E+04 | (EC50)^11)^ | | 1.00E+03 | | 1.15E+01 | 1.44E-02 | | 1.26E-03 |
|  | *Cyclotella meneghiniana* | | growth | | 96h | | 1.00E+04 | (NOEC) ^4)^ | |  | | 1.00E+01 |  | | 1.44E-03 |
|  | *Oryzias latipes* | | feeding behaviour | | 9d | | 1.00E+03 | (EC)^12)^ | |  | | 1.00E+00 |  | | 1.44E-02 |
| Bezafibrate | *Vibrio fischeri* | | luminescence | | 30min | | 1.00E+05 | (NOEC)^13)^ | | 1.00E+04 | | 1.00E+01 | 6.38E-04 | | 6.38E-05 |
|  | *Artemia salina* | | immobilisation | | 48h | | 1.00E+05 | (EC50)^3)^ | |  | | 1.00E+01 |  | | 6.38E-05 |
| **Carbamazepine** | *Cyclotella meneghiniana* | | growth | | 96h | | 1.00E+04 | (NOEC)^4)^ | | 1.00E+03 | | 1.00E+01 | 2.66E-02 | | 2.66E-03 |
|  | *Echinogammarus marinus* | | mortality | | 21d | | 1.00E+01 | (LC50)^14)^ | |  | | 1.00E-02 |  | | **2.66E+00** |
|  | *Oryzias latipes* | | feeding behaviour/swimming speed | | 8/9d | | 6.15E+03 | (EC)^12)^ | |  | | 6.15E+00 |  | | 4.32E-03 |
|  | *Paracentrotus lividus* | | embryo-larval development | | 48h | | 1.00E-02 | (EC50)^15)^ | |  | | 1.00E-05 |  | | **2.66E+03** |
|  | *Diopatra neapolitana* | | regeneration capacity | | 11/18d | | 9.00E+00 | ^16)^ | |  | | 9.00E-03 |  | | **2.95E+00** |
|  | *Hediste diversicolor* | | oxidative stress | | 28d | | 9.00E+00 | ^16)^ | |  | | 9.00E-03 |  | | **2.95E+00** |
| Propranolol | *Cyclotella meneghiniana* | | growth | | 96h | | 9.40E+01 | (NOEC) ^4)^ | | 1.00E+03 | | 9.40E-02 | 1.88E-03 | | 2.00E-02 |
| *0.1 ≤ RQ < 1: medium risk*; | | **RQ ≥ 1: high risk** | |  |  |  | | |  | |  | | |  | |
| [^1)^(Wang et al. 201](#_ENREF_34)9); ^2)^(Ando et al. 2007); ^3)^([Minguez et al. 2016](#_ENREF_56)); ^4)^([Ferrari et al. 2004](#_ENREF_19)); ^5)^(Migliore et al. 1993); ^6)^([Andreozzi et al. 2006](#_ENREF_4)); ^7)^([Steger-Hartmann et al. 1999](#_ENREF_9)); ^8)^([Vandenbergh et al. 2003);](#_ENREF_22) ^9)^([FDA-CDER 1996](#_ENREF_76)); ^10)^(Calleja et al. 1994); ^11)^([Ferrari et al. 200](#_ENREF_19)3); ^12)^([Nassef et al. 2010](#_ENREF_57)); ^13)^([Isidori et al. 200](#_ENREF_26)7); ^14)^([Guler and Ford 2010](#_ENREF_36)); ^15)^([Aguirre-Martinez et al. 2015](#_ENREF_2)); ^16)^([Pires et al. 2016](#_ENREF_66)). | | | | | | | | | | | | | | | |

**Table S12** Environmental risk assessment – WWTP effluent maximum concentrations from Santos et al. (2013) applied to current ERA

| **Compound** | **Organism** | **Endpoint** | **Duration** | **Effect conc. [µg L^-1^]** | | **Assessment factor**^1)^ | **PNEC^1)^**  **[µg L^-1^]** | **WWTP**  effluent-max  **[µg L^-1^]** | **RQmax** |
| --- | --- | --- | --- | --- | --- | --- | --- | --- | --- |
| **Erythromycin** | *Selenastrum capricornutum* | growth |  | 1.03E+01 | NOEC^2)^ | 50 | 2.06E-01 | 1.34E-01 | *6,50E-01* |
|  | *Anabaena cylindrica-NIES 19* | growth | 144h | 3.10E+00 | NOEC^3)^ | 50 | 6.20E-02 |  | **2,16E+00** |
|  | *Oncorhynchus mykiss* | oxidative stress (gills) | 28d | 4.00E-01 | EC^4)^ | 50 | 8.00E-03 |  | **1,68E+01** |
|  | *Oncorhynchus mykiss* | oxidative stress (gills) | 28d | 2.00E-01 | EC^4)^ | 50 | 4.00E-03 |  | **3,35E+01** |
| *Clarithromycin* | *Pseudokirchneriella subcapitata* | growth | 72h | 2.00E+00 | EC50^5)^ | 10 | 2.00E-01 | 4.00E-02 | *2,00E-01* |
| **Sulfamethoxazole** | *Synechococcus leopoliensis* | growth | 96h | 5.90E+00 | NOEC^7)^ | 10 | 5.90E-01 | 1.68E+00 | **2,85E+00** |
| Iopromide |  |  |  | 6.80E+04 | ^1)^ | 10 | 6.80E+03 | 8.50E+01 | 1,25E-02 |
| *Paracetamol* | *Pimephales promelas* | reproduction |  | 1.00E+03 | EC50^11)^ | 1000 | 1.00E+00 | 1.06E-01 | *1,06E-01* |
| **Diclofenac** | *Oncorhynchus mykiss* | cytopathology (liver) | 28d | 1.00E+00 | LOEC^12)^ | 10 | 1.00E-01 | 8.31E-02 | *8,31E-01* |
|  | *Salmo trout f. fario* | histopathohogical | 21d | 5.00E-01 | NOEC^13)^ | 10 | 5.00E-02 |  | **1,66E+00** |
| **Bezafibrate** | *Pimephales promelas* | repoduction |  | 5.30E+03 | EC50^11)^ | 5000 | 1.06E+00 | 6.35E-01 | *5,99E-01* |
|  | *Ceriodaphnia dubia* | growth inhibition | 7d | 2.30E+01 | NOEC^14)^ | 5000 | 4.60E-03 |  | **1,38E+02** |
| **Carbamazepine** | *Ceriodaphnia dubia* | reproduction | 7d | 2.50E+01 | NOEC^7)^ | 10 | 2.50E+00 | 4.96E-01 | *1,98E-01* |
|  | *Cyprinus carpio* | cytopathology (kidney) | 28d | 1.00E+00 | LOEC^12)^ | 10 | 1.00E-01 |  | **4,96E+00** |
|  | *Gammarus pulex* | behaviour |  | 1.00E-02 | LOEC^15)^ | 10 | 1.00E-03 |  | **4,96E+02** |
| *Propranolol* | *Hyalella azteca* | reproduction | 7d | 1.00E+00 | NOEC^16)^ | 10 | 1.00E-01 | 1.06E-02 | *1,06E-01* |
| *0.1 ≤ RQ < 1: medium risk*; **RQ ≥ 1: high risk** | | | | | | | | | |
| ^1)^(Bergmann et al. 2011); ^2)^([Eguchi et al. 2004](#_ENREF_25)); ^3)^([Ando et al. 2007](#_ENREF_3)); ^4)^([Rodrigues et al. 2016](#_ENREF_71)); ^5)^ (Isidori et al. 2005); ^7)^([Ferrari et al. 2004](#_ENREF_29)); ^11)^([Sanderson et al. 2003](#_ENREF_76)); ^12)^[(Triebskorn et al. 2007](#_ENREF_81)); ^13)^[(Hoeger et al. 2005](#_ENREF_38)); ^14)^[(Isidori et al. 2007](#_ENREF_42)); ^15)^([De Lange et al. 2006](#_ENREF_15)); ^16)^([Huggett et al. 2002](#_ENREF_40)). | | | | | | | | | |

**Table S13**: Overview of environmental risk assessment (ERA) studies in water

| **Study** | This study  Portugal **Mondego** River | | | | Figuiere et al. (2022) ^1^  **Sweden surface waters** | Fonseca et al. (2020) ^2^  **Spain** **Mijares** River | Pereira et al (2017) ^3^  **Portugal** **Mondego**, **Tagus** a.o. rivers incl. estuaries | Pereira et al. (2020b) ^3^  Different countries **surface waters** | Biel-Maeso et al. (2018) ^4^  **Spain** **Bay & Gulf of Cadiz** | |
| --- | --- | --- | --- | --- | --- | --- | --- | --- | --- | --- |
| **Study site(s)** | **M1-M11**  Upstream of Coimbra | **M12**  Close to WWTP | **M13-M16**  Downstream of Coimbra | **M18-A01**  Estuary/ Coast | fresh water | fresh water | fresh and brackish water | fresh water | **Cadiz Bay**  fresh-, brackish-, seawater | **Cadiz Gulf**  seawater |
| **Compound** | **RQmax** | | | | **Mean RQ** | **PAF [%]** | **RQ (est. RQmax)** ^5^ | **RQmax** | **RQmax** | |
| Erythromycin | 1.45E-02 | *9.40E-01* | *3.77E-01* | 2.65E-02 | *4.70E-01* | < 0.1 | < 1.00E-01 (> 0.1) | **1 - 150** (A) / *0.1* **- 15** (I) | 0.06 | < 0.01 |
| Clarithromycin | 3.41E-03 | 2.33E-02 | 1.31E-02 | **2.00E+01** | *1.60E-01* | < 0.1-1.13 | *1.0E-01 - 1.0E+00* A (**> 1**) | **1E+01 - 1E+02** (A) | < 0.01 |  |
| Roxithromycin | 6.16E-04 | 1.02E-03 |  |  | *1.30E-01* |  |  |  | < 0.01 |  |
| Sulfamethoxazole | 1.36E-03 | 2.18E-02 | 1.28E-02 | 5.74E-02 | 1.60E-02 | < 0.1-0.88 |  |  | 0.06 |  |
| Sulfadimethoxine | 2.37E-02 | 1.35E-02 | 1.41E-02 | 2.03E-05 |  |  |  |  | < 0.01 |  |
| Tiamulin | 3.27E-02 | 1.93E-02 |  |  |  |  |  |  |  |  |
| Lincomycin | 4.64E-03 | 1.00E-02 | 8.63E-03 | 7.33E-03 |  | < 0.1 |  |  | < 0.01 | < 0.01 |
| Iopromide | 1.04E-05 | 4.13E-04 | 1.81E-04 | 2.24E-03 | 2.60E-02 |  |  |  |  |  |
| Amidotrizoic acid | 4.53E-07 | 3.77E-06 | 2.77E-06 |  | *1.10E-01* |  |  |  |  |  |
| Iohexol |  | 7.05E-05 | 1.01E-05 |  |  |  |  |  |  |  |
| Paracetamol | 4.78E-04 | 4.66E-03 | 2.79E-03 | 2.55E-03 |  | 0.1-0.56 | < 1.00E-01 *(> 0.1*) | *0.02* **- 5** (I) | < 0.01 | < 0.01 |
| Diclofenac | 6.17E-02 | **1.20E+00** | *3.52E-01* | 1.44E-02 | *6.10E-01* | *0.13-9.65* | **1.02E+00** F (**> 1**) | *0.01* **- 10** (A) / **1 - 18** (F) | < 0.01 | < 0.01 |
| Bezafibrate | 2.21E-02 | **2.22E+00** | 8.58E-01 | 6.38E-05 | 3.00E-03 | < 0.1 | < 1.00E-01 *(> 0.1*) | *0.01* **- 1.1** (I) | < 0.01 |  |
| Carbamazepine | **7.57E+00** | **5.26E+01** | **4.22E+01** | **2.66E+03** | 2.80E-02 | < 0.1 | < 1.00E-01 *(> 0.1*) | **1 - 15** (A) / **100 - 1500** (I) | < 0.01 | < 0.01 |
| Primidone | 5.25E-03 | 4.22E-02 | 2.55E-02 |  | 1.20E-03 | < 0.1-0.28 |  |  |  |  |
| Nadolol |  |  |  |  |  |  |  |  | < 0.01 | < 0.01 |
| Propranolol | 8.66E-04 | 1.47E-02 | 4.78E-03 | 2.00E-02 | 1.10E-02 |  |  |  | 0.01 |  |

Low risk; *0.1 ≤ RQ < 1: medium risk*; **RQ ≥ 1: high risk**

^1^ Recipient surface waters upstream & downstream of WWTPs. Lowest effect concentration (out of daphnia, fish, invertebrate) incl. individual assessment factor for PNEC calculation

^2^ Recipient surface waters upstream & downstream of WWTPs. Sampling times: summer, autumn, winter. PAF (Potentially Affection Fraction), Risk calculation using Species Sensitivity Distributions for chronic exposure (bacteria, algae, invertebrates and fish): < 5 %: low risk; 5 % - 25 %: moderate risk; > 25 %: severe risk

^3^ Lowest effect concentration (out of daphnia, fish, invertebrate) incl. assessment factor for PNEC calculation, A: Algae, I: Invertebrate; F: Fish

^4^ “worst case scenario” - lowest effect concentration (out of daphnia, fish, invertebrate) incl. assessment factor of 1000 for PNEC calculation; for marine RQ calculation freshwater species were selected that tolerate brackish environment, low risk

^5^ Estimation for drought periods

# References

Aguirre-Martinez GV, Owuor MA, Garrido-Perez C, Salamanca MJ, Del Valls TA, Martin-Diaz ML (2015): Are standard tests sensitive enough to evaluate effects of human pharmaceuticals in aquatic biota? Facing changes in research approaches when performing risk assessment of drugs. Chemosphere 120, 75-85. <https://doi.org/10.1016/j.chemosphere.2014.05.087>

Ando T, Nagase H, Eguchi K, Hirooka T, Nakamura T, Miyamoto K, Hirata K (2007): A novel method using cyanobacteria for ecotoxicity test of veterinary antimicrobial agents. Environ Toxicol Chem 26, 601-606. https://doi.org/10.1897/06-195R.1

Andreozzi R, Canterino M, Lo Giudice R, Marotta R, Pinto G, Pollio A (2006): Lincomycin solar photodegradation, algal toxicity and removal from wastewaters by means of ozonation. Water Res 40, 630-638. <https://doi.org/10.1016/j.watres.2005.11.023>

Bergmann A, Fohrmann R, Weber FA (2011): Zusammenstellung von Monitoringdaten zu Umweltkonzentrationen von Arzneimitteln. In: UBA-FB 001525, Umweltbundesamt, ISSN 1862-4804, Dessau-Roßlau

Bialk-Bielinska A, Stolte S, Arning J, Uebers U, Boschen A, Stepnowski P, Matzke M (2011): Ecotoxicity evaluation of selected sulfonamides. Chemosphere 85, 928-933. <https://doi.org/10.1016/j.chemosphere.2011.06.058>

Biel-Maeso M, Baena-Nogueras RM, Corada-Fernandez C, Lara-Martin PA (2018): Occurrence, distribution and environmental risk of pharmaceutically active compounds (PhACs) in coastal and ocean waters from the Gulf of Cadiz (SW Spain). Sci Total Environ 612, 649-659. <https://doi.org/10.1016/j.scitotenv.2017.08.279>

Brain RA, Johnson DJ, Richards SM, Sanderson H, Sibley PK, Solomon KR (2004): Effects of 25 pharmaceutical compounds to Lemna gibba using a seven-day static-renewal test. Environ Toxicol Chem 23, 371-382. https://doi.org/10.1897/02-576

Calleja MC, Persoone G, Geladi P (1994): Comparative Acute Toxicity of the First 50 Multicenter Evaluation of in-Vitro Cytotoxicity Chemicals to Aquatic Non-Vertebrates. Arch Environ Con Tox 26, 69-78. https://doi.org/10.1007/BF00212796

Chang XS, Meyer MT, Liu XY, Zhao Q, Chen H, Chen JA, Qiu ZQ, Yang L, Cao J, Shu WQ (2010): Determination of antibiotics in sewage from hospitals, nursery and slaughter house, wastewater treatment plant and source water in Chongqing region of Three Gorge Reservoir in China. Environ Pollut 158, 1444-1450. <https://doi.org/10.1016/j.envpol.2009.12.034>

De Lange HJ, Noordoven W, Murk AJ, Lurling M, Peeters ET (2006): Behavioural responses of Gammarus pulex (Crustacea, Amphipoda) to low concentrations of pharmaceuticals. Aquat Toxicol 78, 209-16. <https://doi.org/10.1016/j.aquatox.2006.03.002>

Eguchi K, Nagase H, Ozawa M, Endoh YS, Goto K, Hirata K, Miyamoto K, Yoshimura H (2004): Evaluation of antimicrobial agents for veterinary use in the ecotoxicity test using microalgae. Chemosphere 57, 1733-1738. <https://doi.org/10.1016/j.chemosphere.2004.07.017>

FDA-CDER (1996): Retrospective review of ecotoxicity data submitted in environmental assessments. FDA Center for Drug Evaluation and Research, Rockville,

MD, USA (Docket No. 96N - 0057)

Ferrari B, Paxeus N, Lo Giudice R, Pollio A, Garric J (2003): Ecotoxicological impact of pharmaceuticals found in treated wastewaters: study of carbamazepine, clofibric acid, and diclofenac. Ecotox Environ Safe 55, 359-370. <https://doi.org/10.1016/S0147-6513(02)00082-9>

Ferrari B, Mons R, Vollat B, Fraysse B, Paxeus N, Lo Giudice R, Pollio A, Garric J (2004): Environmental risk assessment of six human pharmaceuticals: Are the current environmental risk assessment procedures sufficient for the protection of the aquatic environment? Environ Toxicol Chem 23, 1344-1354. https://doi.org/10.1897/03-246

Figuiere R, Waara S, Ahrens L, Golovko O (2022): Risk-based screening for prioritisation of organic micropollutants in Swedish freshwater. J Hazard Mater 429. <https://doi.org/10.1016/j.jhazmat.2022.128302>

Fonseca E, Hernandez F, Ibanez M, Rico A, Pitarch E, Bijlsma L (2020): Occurrence and ecological risks of pharmaceuticals in a Mediterranean river in Eastern Spain. Environ Int 144. <https://doi.org/10.1016/j.envint.2020.106004>

Ginebreda A, Munoz I, de Alda ML, Brix R, Lopez-Doval J, Barcelo D (2010): Environmental risk assessment of pharmaceuticals in rivers: Relationships between hazard indexes and aquatic macroinvertebrate diversity indexes in the Llobregat River (NE Spain). Environ Int 36, 153-162. <https://doi.org/10.1016/j.envint.2009.10.003>

Gros M, Petrovic M, Barcelo D (2009): Tracing Pharmaceutical Residues of Different Therapeutic Classes in Environmental Waters by Using Liquid Chromatography/Quadrupole-Linear Ion Trap Mass Spectrometry and Automated Library Searching. Anal Chem 81, 898-912. https://doi.org/10.1021/ac801358e

Guler Y, Ford AT (2010): Anti-depressants make amphipods see the light. Aquat Toxicol 99, 397-404. <https://doi.org/10.1016/j.aquatox.2010.05.019>

Halling-Sorensen B (2000): Algal toxicity of antibacterial agents used in intensive farming. Chemosphere 40, 731-739. <https://doi.org/10.1016/S0045-6535(99)00445-2>

Hoeger B, Kollner B, Dietrich DR, Hitzfeld B (2005): Water-borne diclofenac affects kidney and gill integrity and selected immune parameters in brown trout (Salmo trutta f. fario). Aquat Toxicol 75, 53-64. <https://doi.org/10.1016/j.aquatox.2005.07.006>

Huggett DB, Brooks BW, Peterson B, Foran CM, Schlenk D (2002): Toxicity of select beta adrenergic receptor-blocking pharmaceuticals (B-blockers) on aquatic organisms. Arch Environ Con Tox 43, 229-235. https://doi.org/10.1007/s00244-002-1182-7

Isidori M, Lavorgna M, Nardelli A, Pascarella L, Parrella A (2005): Toxic and genotoxic evaluation of six antibiotics on non-target organisms. Sci Total Environ 346, 87-98. <https://doi.org/10.1016/j.scitotenv.2004.11.017>

Isidori M, Lavorgna M, Palumbo M, Piccioli V, Parrella A (2007): Influence of alkylphenols and trace elements in toxic, genotoxic, and endocrine disruption activity of wastewater treatment plants. Environ Toxicol Chem 26, 1686-1694. https://doi.org/10.1897/06-320R2.1

Kotke D, Gandrass J, Xie ZY, Ebinghaus R (2019): Prioritised pharmaceuticals in German estuaries and coastal waters: Occurrence and environmental risk assessment. Environ Pollut 255. <https://doi.org/10.1016/j.envpol.2019.113161>

Lolic A, Paiga P, Santos LHMLM, Ramos S, Correia M, Delerue-Matos C (2015): Assessment of non-steroidal anti-inflammatory and analgesic pharmaceuticals in seawaters of North of Portugal: Occurrence and environmental risk. Sci Total Environ 508, 240-250. <https://doi.org/10.1016/j.scitotenv.2014.11.097>

Loos R, Tavazzi S, Paracchini B, Canuti E, Weissteiner C (2013): Analysis of polar organic contaminants in surface water of the northern Adriatic Sea by solid-phase extraction followed by ultrahigh-pressure liquid chromatography-QTRAP(A (R)) MS using a hybrid triple-quadrupole linear ion trap instrument. Anal Bioanal Chem 405, 5875-5885. https://doi.org/10.1007/s00216-013-6944-8

Madureira TV, Barreiro JC, Rocha MJ, Rocha E, Cass QB, Tiritan ME (2010): Spatiotemporal distribution of pharmaceuticals in the Douro River estuary (Portugal). Sci Total Environ 408, 5513-5520. <https://doi.org/10.1016/j.scitotenv.2010.07.069>

Migliore L, Brambilla G, Grassitellis A, Dojmi di Delupis G (1993): Toxicity and bioaccumulation of sulphadimethoxine in Artemia (Crustacea, Anostraca). Int. J. Salt

Lake Res. 2(2), 141-152. https://doi.org/10.1007/BF02905906

Minguez L, Pedelucq J, Farcy E, Ballandonne C, Budzinski H, Halm-Lemeille MP (2016): Toxicities of 48 pharmaceuticals and their freshwater and marine environmental assessment in northwestern France. Environ Sci Pollut R 23, 4992-5001. https://doi.org/10.1007/s11356-014-3662-5

Nassef M, Matsumoto S, Seki M, Khalil F, Kang IJ, Shimasaki Y, Oshima Y, Honjo T (2010): Acute effects of triclosan, diclofenac and carbamazepine on feeding performance of Japanese medaka fish (Oryzias latipes). Chemosphere 80, 1095-1100. <https://doi.org/10.1016/j.chemosphere.2010.04.073>

Nodler K, Voutsa D, Licha T (2014): Polar organic micropollutants in the coastal environment of different marine systems. Mar Pollut Bull 85, 50-59. <https://doi.org/10.1016/j.marpolbul.2014.06.024>

Pereira A, Silva L, Laranjeiro C, Lino C, Pena A (2020): Selected Pharmaceuticals in Different Aquatic Compartments: Part II-Toxicity and Environmental Risk Assessment. Molecules 25. https://doi.org/10.3390/molecules25081796

Pereira AMPT, Silva LJG, Lino CM, Meisel LM, Pena A (2016): Assessing environmental risk of pharmaceuticals in Portugal: An approach for the selection of the Portuguese monitoring stations in line with Directive 2013/39/EU. Chemosphere 144, 2507-2515. <https://doi.org/10.1016/j.chemosphere.2015.10.100>

Pereira AMPT, Silva LJG, Laranjeiro CSM, Meisel LM, Lino CM, Pena A (2017): Human pharmaceuticals in Portuguese rivers: The impact of water scarcity in the environmental risk. Sci Total Environ 609, 1182-1191. <https://doi.org/10.1016/j.scitotenv.2017.07.200>

Pires A, Almeida A, Correia J, Calisto V, Schneider RJ, Esteves VI, Soares AMVM, Figueira E, Freitas R (2016): Long-term exposure to caffeine and carbamazepine: Impacts on the regenerative capacity of the polychaete Diopatra neapolitana. Chemosphere 146, 565-573. <https://doi.org/10.1016/j.chemosphere.2015.12.035>

Rodrigues S, Antunes SC, Correia AT, Nunes B (2016): Acute and chronic effects of erythromycin exposure on oxidative stress and genotoxicity parameters of Oncorhynchus mykiss. Sci Total Environ 545-546, 591-600. <https://doi.org/10.1016/j.scitotenv.2015.10.138>

Ruff M, Mueller MS, Loos M, Singer HP (2015): Quantitative target and systematic non-target analysis of polar organic micro-pollutants along the river Rhine using high-resolution mass-spectrometry - Identification of unknown sources and compounds. Water Res 87, 145-54. <https://doi.org/10.1016/j.watres.2015.09.017>

Sanderson H, Johnson DJ, Wilson CJ, Brain RA, Solomon KR (2003): Probabilistic hazard assessment of environmentally occurring pharmaceuticals toxicity to fish, daphnids and algae by ECOSAR screening. Toxicol Lett 144, 383-395. <https://doi.org/10.1016/S0378-4274(03)00257-1>

Singer H (2009): Multikomponenten-Screening für den Rhein bei Basel- in Zusammenarbeit mit dem Bundesamt für Umwelt BAFU, Abschlussbericht, eawag aquatic

research, Dübendorf

Sadutto D, Andreu V, Ilo T, Akkanen J, Pico Y (2021): Pharmaceuticals and personal care products in a Mediterranean coastal wetland: Impact of anthropogenic and spatial factors and environmental risk assessment. Environ Pollut 271. <https://doi.org/10.1016/j.envpol.2020.116353>

Steger-Hartmann T, Lange R, Schweinfurth H (1999): Environmental risk assessment for the widely used iodinated X-ray contrast agent iopromide (Ultravist). Ecotox Environ Safe 42, 274-281. <https://doi.org/10.1006/eesa.1998.1759>

Steger-Hartmann T, Länge R, Schweinfurth H (1998): Umweltverhalten und ökotoxikologische Bewertung von iodhaltigen Röntgenkontrastmitteln. Vom Wasser 91,

185-194

Triebskorn R, Casper H, Scheil V, Schwaiger J (2007): Ultrastructural effects of pharmaceuticals (carbamazepine, clofibric acid, metoprolol, diclofenac) in rainbow trout (Oncorhynchus mykiss) and common carp (Cyprinus carpio). Anal Bioanal Chem 387, 1405-1416. https://doi.org/10.1007/s00216-006-1033-x

Vandenbergh GF, Adriaens D, Verslycke T, Janssen CR (2003): Effects of 17 alpha-ethinylestradiol on sexual development of the amphipod Hyalella azteca. Ecotox Environ Safe 54, 216-222. <https://doi.org/10.1016/S0147-6513(02)00030-1>

Wang GX, Zhang Q, Li JL, Chen XY, Lang QL, Kuang SP (2019): Combined effects of erythromycin and enrofloxacin on antioxidant enzymes and photosynthesis-related gene transcription in Chlorella vulgaris. Aquat Toxicol 212, 138-145. <https://doi.org/10.1016/j.aquatox.2019.05.004>

Wille K, Noppe H, Verheyden K, Vanden Bussche J, De Wulf E, Van Caeter P, Janssen CR, De Brabander HF, Vanhaecke L (2010): Validation and application of an LC-MS/MS method for the simultaneous quantification of 13 pharmaceuticals in seawater. Anal Bioanal Chem 397, 1797-1808. https://doi.org/10.1007/s00216-010-3702-z
